# Maternal low-intensity psychosocial telemental interventions in response to COVID-19 in Qatar: study protocol for a randomized controlled trial

**DOI:** 10.1186/s13063-021-05339-w

**Published:** 2021-06-07

**Authors:** Sarah Naja, Rowaida Elyamani, Mohamad Chehab, Mohamed Siddig, Abdullah Al Ibrahim, Tagreed Mohamad, Rajvir Singh, Iheb Bougmiza

**Affiliations:** 1 Hamad Medical Corporation, Doha, Qatar; 2 Primary Health Care Corporation, Doha, Qatar

**Keywords:** Maternal mental health, EPDS, COVID-19, Telemental intervention

## Abstract

**Introduction:**

As COVID-19 is spreading, new psychological health problems are suspected to be emerging among pregnant women. Higher maternal mental health distress, including perinatal anxiety, depression, and COVID-19-specific phobia, is supposed to be increasing during the pandemic, which impacts pregnant women’s health and their infants and calls for intervention. Due to the social distancing protocols posed by the pandemic, telemental health interventions have fast become the most common form of psychosocial support for maternal mental health. However, there is no evidence of the effectiveness of maternal low-intensity psychosocial telemental interventions in improving mental health outcomes. The trial’s objective is to evaluate the clinical efficacy of telemental low-intensity psychosocial interventions in pregnant and postpartum women attending the Women Wellness and Research Centre in Qatar in the era of the COVID-19 pandemic.

**Methods and analysis:**

The clinical trial is randomized in which pregnant women will be assigned equally through block randomization between two arms: (1) a control group and (2) an intervention group. The primary endpoint is the perinatal psychological distress, including perinatal depression, anxiety, and COVID-19 phobia in their third trimester; the secondary, tertiary, fourth, and fifth endpoints will be in the postnatal period (3–5 weeks, 2–4 months, 5–7 months, and 8–10 months). This trial involves pregnant women in their second trimester with no mental health illness history who communicate in English and Arabic and consent to participate. A sample size of 58 (29 participants per arm) is targeted.

**Discussion:**

This study will provide recommendations about the efficacy of low-intensity psychosocial maternal telemental interventions to be implemented as a preventive service.

**Trial registration:**

2a-ClinicalTrials.gov NCT04594525. Registered on October 20, 2020.

**Supplementary Information:**

The online version contains supplementary material available at 10.1186/s13063-021-05339-w.

## Administrative information

**Table:** 

**Title {1}**	Maternal Low-Intensity Psychosocial Telemental Interventions in response to COVID-19 in Qatar: A Protocol of Randomized Clinical Trial
**Trial registration {2a and 2b}**	2a-ClinicalTrials.gov Identifier: NCT04594525 https://www.clinicaltrials.gov/ct2/show/NCT04594525
**Protocol version {3}**	Protocol Version: October 20, 2020
**Funding {4}**	Hamad Medical Research Centre fast track MRC-05-087/ registration MRC-01-20-1129.
**Author details {5a}**	(1): Hamad Medical Corporation, Doha, Qatar; snaja1@hamad.qa, RElyamani@hamad.qa, mchehab2@hamad.qa, mahmed85@hamad.qa, AAllbrahim3@hamad.qa, tmohammed1@hamad.qa. rsingh@hamad.qa. (2): Primary Health Care Corporation, Doha, Qatar; mbougmiza@phcc.gov.qa *Correspondence: Dr. Sarah Naja, Community Medicine Residency Program, Department of Medical Education, Hamad Medical Corporation, Doha, Qatar. P.O. Box 3050; dr.sj.naja@gmail.com Dr. Sarah Naja: Principle Investigator: Community Medicine, Hamad Medical Corporation, Doha, Qatar, dr.sj.naja@gmail.com
**Name and contact information for the trial sponsor {5b}**	HMC Institutional Review Board (HMC-IRB) Chair at 55546316 HMC-IRB Office at 40256410 (from Sunday to Thursday between 7:00am-3:00pm) or email at irb@hamad.qa
**Role of sponsor {5c}**	Hamad Medical Corporation provided support in salaries for authors [SN, RE, MC, MS, AA, TM, RS]. Still, it did not have any additional role in the study design, data collection, analysis, decision to publish, or manuscript preparation.

## Introduction

### Background and rationale{6a}

The infectious outbreak of the novel coronavirus was first identified in Wuhan City, China, and reported to the World Health Organization (WHO) on December 31, 2019. COVID-19 caused by severe acute respiratory syndrome coronavirus 2 (SARS-CoV-2) has been widely publicized as “killer pneumonia” transmitted via droplets and fomites during close unprotected contact between an infector and infected with no precise treatment [1]. The life-threatening aspect of COVID-19 was vividly described by the media, constantly alarming people of the dangers of this disease. In addition to the actual physical health danger posed by the virus, the pandemic has also impacted mental health [2].

Perinatal depression and anxiety are increasingly recognized as crucial public health issue [3]. Emerging infectious outbreak decreases social support and fuels perinatal mental health disorders. For instance, a previous infectious outbreak revealed that pregnant women tend to overestimate the risk of contracting respiratory illness and report higher anxiety levels [4]. Recent data show a higher prevalence of psychological distress (anxiety and depression) in pregnancy during the COVID-19 pandemic across several countries [5–7].

During the pandemic, the burden of perinatal health problems reported in Qatar was higher than before the pandemic; perinatal depression increased by 10% (24% vs. 34.4%), and perinatal anxiety increased by 22.8% (16.4% vs. 39.2%). These studies reported the prevalence based on screening tools and utilized similar conceptual definitions in distinguishing depressed and anxious pregnant women from those without outcome [8–10]. The alarming rise in perinatal mental health distress may negatively affect the expecting mothers and their offspring leading to an increase in the infants’ risk of pre-term delivery and neurodevelopmental disorders [11]. Crucial interventions are needed to address perinatal psychological distress among Qatar’s maternal population during the pandemic.

Most guidelines recommend psychological or behavioral treatments as first-line therapy for pregnant women with mild to moderate depression [12], considering it an essential strategy for mitigating postnatal depression and preventing behavioral and developmental problems in upcoming children [13, 14], supported by the United States Preventive Services Task Force (USPSTF) recommendation [15].

In infectious pandemics and emergencies, telemental health is a practical strategy that reaches the population [16, 17]. The outcome of systematic analysis of RCTs (randomized controlled trials) showed the effectiveness of telemental service in the general population, leading to a significant reduction in anxiety, depressive symptoms, distress, and fear [18]. However, no substantial evidence on the effectiveness of Telemental health intervention among the pregnant population.

### Objectives {7}

The objectives are as follow: to assess the impact of telemental low-intensity psychosocial interventions on perinatal mental health outcomes, through assuming the superiority of the telemental health intervention on the control arm in improving perinatal psychological distress among the perinatal population, and working on rejecting the null hypothesis stating that pregnant women receiving low-intensity psychosocial interventions have the same psychological distress (perinatal depression, perinatal anxiety, and COVID-19 phobia) compared to pregnant women not receiving the intervention during the COVID-19 pandemic.

The following research questions will be addressed:

1. What is the effect size of telemental health on perinatal mental health outcomes?
2. What are the period prevalence and incidence of perinatal depression, anxiety, and COVID-19 phobia among perinatal women during the COVID-19 pandemic in Qatar?
3. What other factors influence perinatal mental outcomes during the COVID-19 pandemic in Qatar?

### Trial design {8}

A randomized controlled, parallel trial of the effect of low-intensity psychological maternal telemental health intervention on perinatal psychological outcomes among pregnant and postpartum women.

The study will be carried in two phases.

#### Phase one: training of psychologists

The investigators will train two psychologists that are English and Arabic speakers on the WHO materials [19]. Consequently, the data collectors will be prepared to undertake the approved research, motivate interviewers, and ensure the good overall quality of data. The training includes both formal classroom training and hands-on experience with telemedicine techniques.

#### Phase two: screening and enrolling participants

The investigators will follow the Consolidated Standards of Reporting Trials (CONSORT) in displaying steps of recruitments and randomization. The eligible participants will be selected randomly from the registered pregnant women at Women Wellness and Research Centre (WWRC) and will be contacted by phone to inform the study through verbal informed consent.

**Fig. 1:**
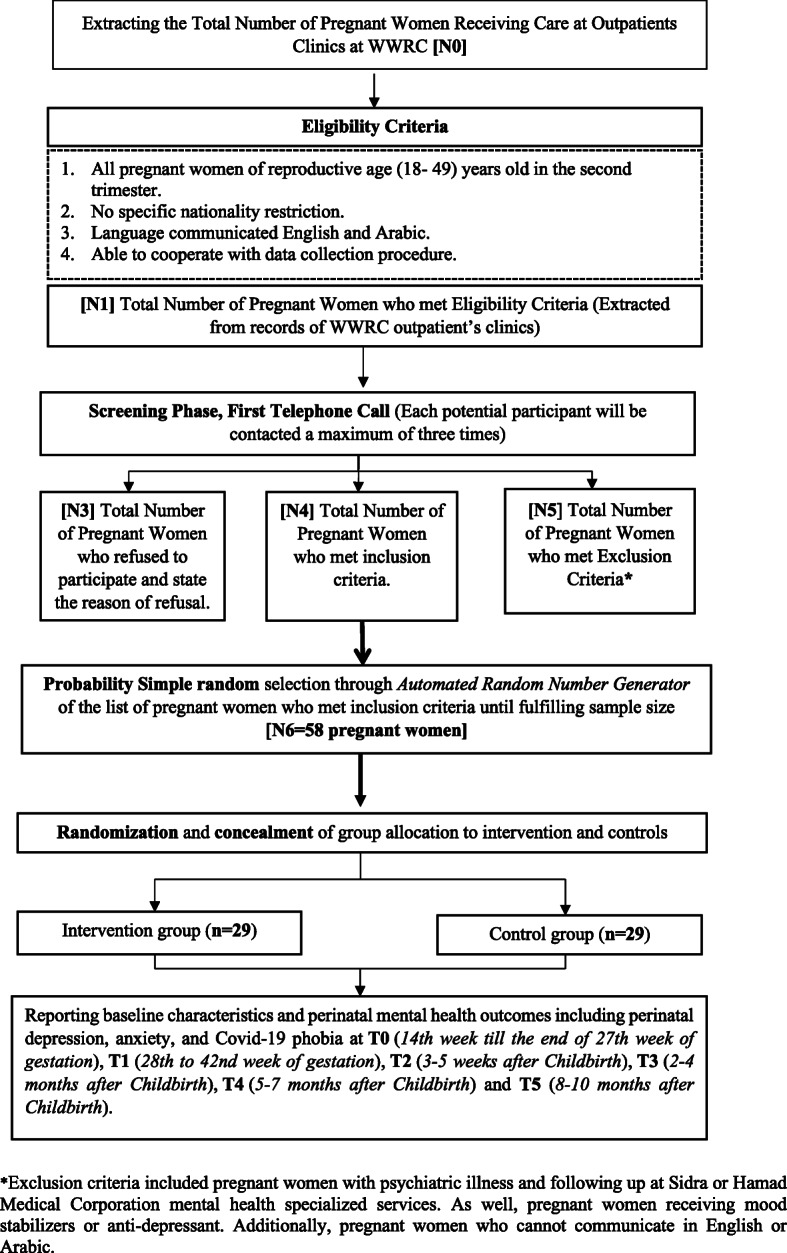
Study flow

Randomization (1:1) with an automated online system ensuring that the research team will not affect randomization. Participants will complete all the assessments via telephone interviewing but following a prewet transcript, and therefore, their responses could be affected by the trial team. A cohort of participants will be followed from the time of approval of the study until the end of interventional sessions (T5-session 6), approximately 1 year after the baby is born. Figure 1 summarizes the study flow.

## Methods: participants, interventions, and outcomes

The manuscript is written in accordance with the Standard Protocol Items: Recommended items to address in a clinical trial protocol and related documents (SPIRIT) guidelines.

### Study setting {9}

The WWRC is the primary setting for screening and recruiting the participants. The WWRC is a paramount governmental health facility that provides continuous health services for women during their reproductive age, following them from antenatal to postnatal. The center accommodates 17,000 births per year. The WWRC is highly accessible and affordable by the country’s population. WWRC’s patient cohort offers a good representation of the whole community. The average number of patients attending each clinic can reach 20 per shift. WWRC’s antenatal clinic participation rate was as high as 70% of the total live births during 2019 (about 12,896 pregnant women) [20].

### Patient recruitment and study procedures

#### Eligibility criteria {10}

##### Patient inclusion and exclusion criteria

The target population is perinatal women following up at the Women’s Wellness and Research Centre (WWRC). The inclusion criteria include all pregnant women aged 18 and above in their second trimester who agree and consent to receive teleconsultation. Participants need to communicate in English and Arabic. However, there is no specific nationality restriction. We will exclude pregnant women who cannot download the Virtual Single Execution Environment (VSee) application and those with psychiatric illness. Furthermore, pregnant women receiving mental health medications and following up at Sidra hospital or Hamad Medical Corporation mental health services will be excluded.

#### Who will take informed consent? {26a}

The consent will be taken by delegates’ obstetrics and gynecology physicians of the approved representatives from WWRC in the screening phase through the phone. In the data collection phase, the psychologists will take consent at the beginning of the video call prior to the interview.

#### Additional consent provisions for collection and use of participant data and biological specimens {26b}

No biological specimen is required in the study; it is a psychosocial intervention. Consents are available upon request.

#### Plans to promote participant retention and complete follow-up {18b}

For adherence to the intervention, participants in both groups will be contacted by phone every 3 weeks.

For adherence to questionnaires, psychologists will collect the data through interviews, less commonly to have missing data. In case of missing data, study participants will be contacted and reminded about the completion by a psychologist through telephone.

#### Patient withdrawal

Study participants may leave the study at any time and may withdraw consent to study participation without negative consequences. The reasons for discontinuation will be asked in the consent form and will be recorded.

### Interventions

#### Explanation for the choice of comparators {6b}

The WHO low-intensity psychosocial intervention “healthy thinking” is to be provided through platform VSee. The comparative arm is selected from the same setting to prevent any selection bias.

#### Intervention description {11a}

##### Intervention group (IG)

This a series of Low-Intensity Psychological Interventions based on the healthy thinking manual. The Thinking Healthy approach attends to giving attention to overall maternal mental well-being by delivering a simplified form of cognitive behavior therapy (CBT) recommended by the Mental Health Gap Action Programme (mhGAP) [19]. It will be tailored and delivered through six video conferencing sessions, real-time interaction between patients and therapists via VSee video connection. Each session is a one-to-one session and may last up to 45 min. Adherence will be monitored and documented on logbooks and medical records. Details about the sessions are available in the WHO manual.

##### Control group (CG)

Participants in the CG will be screened for outcomes via the same platform in addition to usual care.

#### Criteria for discontinuing or modifying allocated interventions {11b}

Participants can leave the study at any time and may withdraw consent. There will be no criteria for discontinuing or modifying allocated interventions.

#### Strategies to improve adherence to interventions {11c}

The data collectors will ensure the participants’ adherence in both groups through phone reminders about their appointment and the flexible, convenient booking of their meetings.

#### Relevant concomitant care permitted or prohibited during the trial {11d}

In severe depression or suicidal ideation during the study, the participant will be referred to urgent psychiatric care based on HMC Guidelines and receive appropriate treatment.

#### Provisions for post-trial care {30}

There is no anticipated harm, and investigators will not provide compensation for trial participation. The participants identified in the trial suffering from maternal mental distress will be receiving care, follow-up, and treatment from psychiatry based on HMC Guidelines.

### Participant timeline {13}

Prospectively, the participants will be followed for approximately 1 year (from their second trimester to around 8 to 10 months postnatal). Participants will be enrolled and assessed following the Standard Protocol Items: Recommendations for Intervention Trials (SPIRIT) schedule as shown in Table 1.

**Table 1:** SPIRIT schedule

	Study period						
	Screening	Allocation	Post-allocation				
	Pregnancy		Pregnancy		Postpartum		
Timepoint**	***T-***_***1***_	**0**	***T***_***0***_	***T***_***1***_	***T2***	***T3***	***T5***
Eligibility screen	X						
Informed consent	X		X	X	X	X	X
Randomization		X					
Interventions:							
				X	X	X	X
Telemental low-intensity psychosocial interventions A							
Control Group B				X	X	X	X
Assessments:							
1. Mental health history	X						
2. Medication use	X						
3. COVID-19 status			X	X	X	X	X
4. Perinatal depression			X	X	X	X	X
5. COVID-19-specific phobia			X	X	X	X	X
6. Perinatal anxiety			X	X	X	X	X
7. Sociodemographic characteristics			X	X	X	X	X
8. Pregnancy-related factors			X	X			
9. Acute medical problem			X	X	X	X	X
10. Previous maternal and neonatal complication			X	X			
11. Chronic illnesses			X	X			
12. Behavioral factors			X	X	X	X	X
13. Stressful life events			X	X	X	X	X
14. Newborn health					X	X	X

*T*_*−1*_ before enrolment it is the screening phase in the 2nd trimester [14th week until the end of 27th], *T*_*0*_ after randomization and allocation it is baseline assessment [14th week until the end of 27th], *T*_*1*_ is during the 3rd trimester [28th week until the end of 42nd week] the first intervention session, *T*_*2*_ during early postpartum 3–5 weeks, *T*_*3*_ in 2–4 months postpartum, *T*_*4*_ in 5 to 7 months postnatal, *T*_*5*_ in 8 to 10 months postnatal

### Outcomes {12}

#### Primary outcomes

The primary outcomes are baseline (T0) scores of perinatal depression, perinatal anxiety, and COVID-19-specific phobia during late pregnancy and during the follow-up up to 10 months postpartum, additionally computing the changes in the scores from the baseline [(T1–T0), (T2–T1), (T3–T2), (T4–T3), and (T5–T4)].

We will evaluate the primary outcomes through video conferencing sessions via VSee video connection based on standard tools.

Edinburgh Postnatal Depression (EPDS-10) will be used to assess perinatal depression. The scale showed to be a reliable screening tool in Qatar [9]. Cumulative evidence demonstrated a pooled sensitivity of 0.80 and a pooled specificity of 0.81 [21].

The anxiety subscale of EPDS-3A will be used to assess perinatal anxiety. It is identified to have acceptable sensitivity and specificity based on a systematic review of perinatal anxiety tools [22].

Corona-specific phobia, a severe fear specific to the emerging virus, will be assessed through the Corona-Specific Phobia questionnaire that consists of a seven-item scale. Answers are based on the five-item Likert-type scale, and it showed internal consistency (*α* = 0.82) with robust psychometric properties. A total score is calculated by adding up each item score (ranging from 7 to 35). The higher the score, the greater the fear of coronavirus-19 [23].

#### Secondary outcomes

A structured interview will be communicated through video conferencing sessions via VSee video connection to evaluate secondary outcomes that may act as independent variables or confounders. The structured interview includes domains (sociodemographic, pregnancy-related characteristics, current medical problems in pregnancy, corona status, medications, previous maternal and neonatal complications, chronic diseases, mental history, behavioral factors, and possible psychosocial stresses).

### Statistical methods {14}

The required sample size was calculated using the sample size calculation OpenEpi® software version 3.01 available online [24]. In testing the null hypothesis at a 95% level of confidence interval (CI), the error rate of 5% with power = 80%. We used the following formula for sample size calculation:

$$ \mathrm{n}1=\left(\mathrm{Z}\upalpha /2+\mathrm{Z}\upbeta \right)2\ast \left(\mathrm{p}1\left(1-\mathrm{p}1\right)+\mathrm{p}2\left(1-\mathrm{p}2\right)\right)/\mathrm{r}\left(\mathrm{p}1-\mathrm{p}2\right)2 $$

and

n2 = r*n1

where:

n1 = number of exposed (intervention group)

n2 = number of unexposed (control group)

Zα/2 = standard normal deviate for the two-tailed test based on alpha level (relates to the confidence interval level) [alpha = 0.05]

Zβ = standard normal deviate for the one-tailed test based on beta-level (relates to the power level) [beta = 0.2]

r = ratio of unexposed to exposed = 1

p1 = proportion of exposed with the outcome: incidence in intervention group: 34% (we used results from two studies that showed and an improvement between 34 and 69% after delivering the interventions to pregnant women) [18, 25].

p2 = proportion of unexposed with the outcome: incidence in the control group: 70% (since no similar studies have been conducted locally; hence, the estimate p1 was set to 70%).

The sample size needed for each group would be 29 pregnant women, and the total would be 58 participants.

### Recruitment {15}

A list of pregnant women in the second trimester who speaks English and or Arabic will be requested. Their contact numbers and the total number and percentage of pregnant women registered at WWRC will be identified at the time of recruitment and during the study period.

### Assignment of interventions

#### Sequence generation {16a}

Probability simple random selection will be selected randomly through the Automated Random Number Generator participants. The obstetric team on the delegation list will screen participants to identify those who fulfill inclusion criteria; then, they will be contacted through phone to consent; this step will continue until the calculated sample size is fulfilled. Each potential participant will be contacted a maximum of three times before withdrawing their name from the potential participant list.

The list of participants who consented to participate will be identified (codes only) with their phone number and contacted to arrange the teleconsultations sessions (six-video sessions) at their convenient time.

#### Concealment mechanism {16b}

Random allocation of the participants will be done into the intervention and control groups. Concealment of group allocation will be done to minimize selection bias. Before commencing the first session, one investigator will randomly allocate eligible participants into two groups. Each participant’s contact details will be written on a printed paper and sealed in an envelope marked with either letter A or B.

#### Implementation {16c}

At the implementation level, another investigator will follow-up with the sessions. The investigator will also hand equal numbers of envelopes to each psychologist randomly. Each psychologist will then open the envelope and contact the patient to begin the teleconsultation session. Patients will give their consent verbally over the phone prior to any teleconsultation sessions.

### Assignment of interventions: blinding

#### Who will be blinded {17a}

Blinding will be done for a data analyst to reduce performance and ascertainment bias. Blinding to data entry will be established by providing a unique code to each subject before entering the analysis data.

#### Procedure for unblinding if needed {17b}

We do not assume that unblinding will be necessary due to the nature of the intervention.

### Data collection and management

#### Plans for assessment and collection of outcomes {18a}

Data will be obtained at different time points using paper-based questionnaires. In the screening phase, the obstetrics and gynecology team will obtain verbal informed consent through the phone.

In the recruitment phase, the trained psychologists will contact participants via phone to book the video interview’s date and time. Psychologists will secure consent before each video conference.

Structured interview was tested for its face, translation, and content validity by expertise (content validity 90%).

If question 10 in the EPDS-10 showed suicidal intentions, the patient would be directed to the emergency department to one of the HMC hospitals or call the hotline to get an urgent referral to assess mental health services staff corporate policies and guidelines.

#### Data management {19}

Data will be entered and analyzed using the Statistical Package for Social Sciences SPSS® V22.0. All electronic and paper-based data will be stored at the Institute of HMC for a maximum period of 10 years and subsequently destroyed.

#### Confidentiality {27}

All patient-related information will be coded to secure patient protection. For trial analyses, validity-checked data will be transferred in the same way to the study statistician in HMC. The identifiable patient information will be password protected. The code identification list linking the subject’s identity should be maintained confidentially and stored separately in a sealed envelope and kept in a locked cabinet along with the study files with limited access. The team should ensure that screening, enrolment, and randomization details are recorded on a screening/enrolment and randomization log.

### Plans for collection, laboratory evaluation, and storage of biological specimens for genetic or molecular analysis in this trial/future use {33}

Not applicable as no biological specimens were collected as part of this trial.

### Statistical methods

#### Statistical methods for primary and secondary outcomes {20a}

Quantitative variables will be described as mean and standard deviations, whereas the frequency with percentages will be calculated for categorical variables. Both the intervention group and control groups will be described in a table with all the characteristics. Chi-square test will be applied to compare percentages of categorical variables. Regression analysis will be carried to stratify data to eliminate potential confounders and effect modifiers. P value 0.05 (two-tailed) was considered for statically significant level.

We will analyze each continuous outcome with a linear mixed-effects regression model to account for the repeated measures over time. We will analyze binary outcomes with a logistic mixed-effects model. Mixed-effects models are the recommended statistical technique for analyzing clinical trials when outcomes are collected at repeated time points. This trial included outcome data at gestation weeks 14 to 40, 3 to 5 weeks postnatal, 2 to 4 months postpartum, 5 to 7 months, and 8 to 10 months postpartum or postnatal available for all participants who will be randomly assigned. The logistic mixed-effects model has the advantage of implicitly accounting for data missing at random. The estimated (adjusted) effect differences from these analyses therefore will be reported. The linear mixed-effects models will include the response variable, timepoint, randomized group, and baseline score as fixed effects. We will model an interaction between time and the randomized group as a fixed effect to estimate treatment effect at all timepoints. Gestational age and postpartum weeks will be included as covariates in the model. We will calculate standardized effect sizes with Cohen’s d.

#### Interim analyses {21b}

There will be no planned interim analysis that would require any adjustment of the significance level (critical value).

#### Methods for additional analyses (e.g., subgroup analyses) {20b}

In subgroup and regression analyses, effects of age, gestational age, and level of education will be explored.

#### Methods in analysis to handle protocol non-adherence and any statistical methods to handle missing data {20c}

All participants will be analyzed in the group they were randomized to following the intention-to-treat principle. Early study discontinuation will be treated as an independent right censoring in the primary analysis. In case of substantial or differential study discontinuations, the validity of the independent censoring assumption will be explored in shared random-effects models of the primary endpoint and time to study discontinuation. Multiple imputation models will be applied in case of missing data. For the knowledge questionnaires, we will follow questionnaire-specific guidance to impute missing data. All details of the statistical analyses, including definitions of analysis populations, will be prespecified in the statistical analysis plan.

#### Plans to give access to the full protocol, participant level-data, and statistical code {31c}

Information will be provided on request.

### Oversight and monitoring

#### Composition of the coordinating center and trial steering committee {5d}

The Institutional Review Board (IRB) or Independent Ethics Committee (IEC) is a body whose primary purpose is to safeguard the rights, safety, and well-being of human subjects involved in research activities conducted under its authority. The HMC Institutional Review Board will be responsible for overseeing monitoring, which is delegated to Clinical Research Monitor(s) (CRM). The HMC IRB office will appoint the CRM. CRM(s) are responsible for site initiation visits and monitoring visits. Monitoring is arranged in line with the IRB study-related risk assessment. The monitor(s) will be familiar with the study protocol and all study procedures before conducting the visits. Initial site training, routine, and close-out monitoring will be planned and conducted throughout the life span of the study. The first monitoring visit will occur after 20 first subjects are enrolled. Consecutive visits will occur at the end of the study. The study will be conducted in full conformance with principles of the “Declaration of Helsinki,” Good Clinical Practice, and within the laws and regulations of the Ministry of Public Health in Qatar, respecting autonomy, justice, and informed consent and fast track ethical approval under protocol number (MRC-05-087) and electronic registration (MRC-01-20-1129). Accepting or rejecting to participate will not affect the usual patient care.

#### Composition of the data monitoring committee, its role and reporting structure {21a}

There will be a formal external independent monitoring by MRC ethical committee. The monitoring includes site visits to the study setting to review informed consents and perform remote checks of Cerner’s database and documentations.

#### Adverse event reporting and harms {22}

The mitigation plan total score showed to be 24, indicating low risk, and the risk assessment showed that the benefits outweigh the risk. According to corporate policies and guidelines, women who will show suicidal thoughts will get an urgent referral to be assessed by mental health services staff.

#### Frequency and plans for auditing trial conduct {23}

The first monitoring visit will occur after 20 first subjects are enrolled. Consecutive visits will occur at the end of the study. During the monitoring visits, the monitor(s) will review 50% of data collection sheets and 50% telephone scripts. After each monitoring visit, the CRM will debrief the study team, i.e., comment on them for things done right and highlight areas that need improvement. At the close-out visit, the monitor(s) will ensure that all queries are resolved, the study product is accounted for and returned or destroyed, and study documents are properly archived.

#### Plans for communicating important protocol amendments to relevant parties (e.g., trial participants, ethical committees) {25}

Approval for protocol modifications and amendments will be sought from the ethical committees at HMC. All changes will be noted in the study registration.

#### Dissemination plans {31a}

This study protocol and study results will be published in major journals to disseminate the study results. Authorship will be shared between persons involved in the study following the International Committee of Medical Journal Editors (ICMJE). Professional writers and persons not directly involved in the writing will not be granted authorship.

## Discussion

To our knowledge, this study is the first research to examine the effectiveness of maternal low-intensity psychosocial telemental interventions in preventing perinatal distress in response to COVID-19 in Qatar.

Cumulative evidence showed that individual psychosocial interventions were more effective than group interventions in preventing and treating perinatal depression. Specifically, face-to-face CBT revealed a protective effect (OR 0.64; 95% CI [0.53–0.75]) and a preventive effect (OR 0.39; 95% CI [0.17–0.60], p 0.001) [26]. Therefore, the regional body of research agreed on the beneficial effects of face-to-face psychosocial interventions. However, there is a lack of studies concerning the effect size of the telemental health approach on perinatal mental health an identified gap to be tested.

The results from this study will show whether maternal low-intensity psychosocial telemental interventions is preventive or not and whether it is recommended to be part of the antenatal package delivered to pregnant women during the COVID-19 pandemic.

One of this study’s strengths is at the level of methodology; applying randomization as a strategy could deal with bias and determine the effectiveness of an intervention in a reliable approach [27]. The prospective nature of the design ensures the temporality between variables and preserves the causation effect. Utilizing a probability sampling technique preserves the external validity of the study. Furthermore, several strategies were employed to decrease the measurement bias, which included extensive training, blinding, and selecting reliable screening tools for assessment. Moreover, it will provide valuable insights into the acceptability of telemental interventions among pregnant women in Qatar, but it is not without limitations. The sample size is not big enough, limiting the generalization of results, and loss of follow-up could be a challenge.

## Trial status

Not recruiting yet. Protocol version: October 20, 2020. It is estimated to start recruiting in June 2021, and recruitment will be completed in June 2021.

## Supplementary Information

**Additional file 1.** Data collection sheet.

**Additional file 2.** Screening sheet.

**Additional file 3.** Consent form.

## Data Availability

Access to the final trial dataset will be limited to the authors and sponsor of the study.

## Abbreviations

WHO: World Health Organization
COVID-19: Coronavirus disease 2019
USPSTF: United States Preventive Services Task Force
SARS-CoV-2: Coronavirus severe acute respiratory syndrome coronavirus 2
MRC: Medical Research Centre
RCTs: Randomized controlled trials
WWRC: Women Wellness and Research Centre
VSee: Virtual Single Execution Environment
EPDS: Edinburgh Postnatal Depression Scale
SPSS: Statistical Package for Social Sciences
CBT: Cognitive behavioral therapy
mhGAP: Mental Health Gap Action Programme
CRM: Clinical Research Monitor
ICMJE: International Committee of Medical Journal Editors
OR: Odds ratio
CI: Confidence interval
