# Supplementary material for: Maternal low-intensity psychosocial telemental interventions in response to COVID-19 in Qatar: study protocol for a randomized controlled trial
Source: Trials. 2021 Jun 7;22:382. doi: 10.1186/s13063-021-05339-w (PMC8181539; doi:10.1186/s13063-021-05339-w)
Supplement: Supplementary file 1 — Additional file 1. Data collection sheet. [file 13063_2021_5339_MOESM1_ESM.pdf]

Structured Questionnaire for T0 *(the participant is pregnant -14 to 42 weeks)*

Appendix III

Structured Questionnaire- Video telecommunication

تاريخ جمع البيانات	عدم المشاركة	اسم جامع البيانات	رقم المشاركة
Date	Drop out	Data Collector Name	Participant Number
………/……. / ……	....	....	....

((بما انك حامل وصحتك تهمنا نتمنى ان ناخذ من وقتك خمسة عشر دقيقة لنسالك بعض الاسئلة .القسم الأول سيشمل مقابلة بحيث يسالك الباحث بعض الاسئلة. اما القسم الثاني فتقرير ذاتي تمليه المشتركة .))

As you are pregnant and your health status matters to us, we would like to ask you some questions. It will include face-to-face interview-based questionnaire and standard tools through the video telecommunication. It will not take more than 15 minutes.

Section I: Questionnaire / الجزء الاول: الاستبيان

يستند هذا الاستبيان على مقابلة تقوم بالتحقيق في بعض الجوانب المتعلقة بحياتك الاجتماعية والديموغرافية والاقتصادية. كما ستسالين عن تاريخك الطبي وعادات أسلوب حياتك ودعم زوجك والمساعدة التي تتوقعينها بعد الولادة.

This is an interview-based questionnaire, going to investigate some aspects related to socio demographical factors, economic factors, medical history and life style habit. Additionally, you will be asked about your partner and the expected postpartum support .

MRC-01-20-1129 Validity: 04 04 2020 - 12 07 2020 E-stamped 05 Apr 2021

ا-العوامل الاجتماعية الديموغرافية والاقتصادية

A- Socio-demographical and economic factors

1. كم عمرك؟ (بالسنوات الكاملة............)

Age (full years.........)

2. ما هي جنسيتك؟.........

Nationality:

3. ما هو مستواك التعليمي؟

What is your highest level of Education?

☐تعليم عالي	☐تعليم ثانوي	☐تعليم ابتدائي	☐لم احظى بتعليم ممنهج
(3) Higher education	Secondary education (2)	(1) Primary education	Didn't attend school (0)

4. عدد الأشخاص الذين يشاركونك نفس المنزل بالإضافة لاولادك ولحضرتك:
....

Total number of members living in your house including your children and yourself: ....

5. هل لديك وظيفة؟

Are you currently employed?

☐نعم موظفة

(1) Yes, Employee

☐كلا ربه منزل

(0) No, Housewife

6. ما هوالدخل الشهري للاسرة؟

Your monthly family income is

☐اكثر اوما يساوي 20001 ريال قطري	☐بين 10001-20000 ريال قطري	☐اقل اوما يساوي 10000ريال قطري
Equal or more than 20001 QR (2)	10001-20000 QR (1)	Up to 10000 QR (0)

B-Current Pregnancy History(ب– تاريخ الحمل الحالي (سيتم مراجعة السجل الصحي

7. هل هذا حملك الأول؟

Is this your first pregnancy? (Gravida)

Yes, if skip to Q10 ☐نعم (1) Yes ، انتقلي الى السؤال 10 ☐لا (0) No

8. هل سبق لك ان خضعتي للولادة؟

Did you gave birth before(Parity)?

MRC-01-20-1129 Validity: 04 04 2020 - 12 07 2020 E-stamped 05 Apr 2021

Yes, if skip to Q12 12 انتقلي الى السؤال ، (1) Yesنعم☒ (0) No لا☐

(1)Yes نعم☐

9. كم طفل لديك؟

………………..

How many children do you have?

……………………………

10. انت حامل في كم اسبوع؟

For how many weeks are you pregnant?

☐بالأسابيع ………….. ☐اخر دورة……….

(Weeks) ………. LMP by patient...

11. هل خططتي مسبقا لهذا الحمل/ الحمل مقصود: من خلال إيقاف وسائل منع الحمل أو اتباع السلوك الذي يحفز على الحمل؟

Did you planned (intended) this pregnancy through stopping contraception or adopting preconception behaviour?

(1)Yes نعم☐ (0)No لا☐

12. هل تريدين هذا الحمل (ستكملين هذا الحمل ولا تتمنى الاجهاض)؟

Do you want this pregnancy (wanted pregnancy)? (I will continue this pregnancy and I am not wishing for abortion)

(1) Yes نعم☐ (0) No لا☐

13. هل عانيت في السابق من العقم (عدم القدرة على الحمل بشكل طبيعي لأكثر من سنة)؟

Did you suffer previously from infertility (inability to conceive spontaneously for more than 1 year despite having regular intercourse)?

☐ Yes نعم (1) ☐ Noلا (0)

14. هل حدث هذا الحمل بشكل طبيعي؟

Currently, did you conceive spontaneously?

☐ نعم تم بشكل طبيعي من دون ادوية او علاج Spontaneously without any medication (0)

☐ لا تم من خلال ادوية Induced through medications (1)

Institutional Review Board Hamad Medical Corporation · Doha · Qatar

MRC-01-20-1129 Validity: 04 04 2020 - 12 07 2020 E-stamped 05 Apr 2021

15. هل تشتكين من اي عوارض صحية؟
Do you have any health complain?
Yes (1)/ نعم -No(0)/كلا

☐ Fatigue/ارهاق

☐ Back pain/الم في الظهر
☐ Vomiting/تقيىء شديد
☐ Headache/ صداع الراس
☐ Sleep disturbance/ اضطراب النوم

16. خلال حملك الحالي هل تم تشخيصك بأي من الامراض الصحية المرتبطة بالحمل ؟
In your current pregnancy, have you been diagnosed with any pregnancy related health Problem/s?

☐ Hyperemesis Gravidarium تقيىء شديد
☐ Gestational Diabetes/ سكر الحمل
☐ Abnormal foetal ultrasound سونار الجنين غير طبيعي…….
☐ مشاكل صحية اخرى..................................
☐ High blood pressure related to pregnancy/ضغط مرتفع مرتبط بالحمل
☐ Anaemia in pregnancy فقر الدم في الحمل
Less than 3month/ اقل من ثلاث اشهر

Suspected Corona ☐

Confirmed Corona ☐

ث -تاريخ الحمل السابق والولادات السابقة(غير مطابقة للمراة الحامل لاول مرة تخطي لسؤال22)
C- Previous Obstetric Problems (Not applicable for Primigravida shift to Q22)

17. الولادة الاخيرة :كانت طبيعي ام قيصري؟
Last delivery was normal or C-section?
☐ Normal Delivery/طبيعي
☐ C-Section/قيصري

18. هل تم تشـخيصـك بأي من الامراض الصـحية أثناء الحمل الاخير او الولادة السابقة؟
Have you been diagnosed with any pregnancy related problem/s during your

MRC-01-20-1129 Validity: 04 04 2020 - 12 07 2020 E-stamped 05 Apr 2021

previous pregnancy or after your last delivery?

☐ولادة مبكرة (قبل سبعة وثلاثين أسبوع)<br>Preterm birth<br>(thirty-seven weeks ago)	☐حمل خارج الرحم<br>Ectopic pregnancy	☐ارتفاع ضغط الدم بسبب الحمل<br>Hypertension due to pregnancy	☐سكر الحمل<br>Gestational Diabetes
☐ ولادة بمساعدة ملقط اوشافط<br>Instrumental delivery	☐اكتئاب ما بعد الولادة<br>Postpartum depression	☐ دخول مستشفى (لغير الولادة)<br>Admission to the hospital (reason different from giving birth)	☐نتائج الموجات فوق الصوتية غير طبيعية...........<br>Ultrasound results are ...abnormal<br>…….
☐ لا يوجد لدي اي مشاكل صحية<br>None	☐ مشاكل صحية اخرى الرجاء التحديد…<br>Other	☐ التهاب بعد الولادة استدعى العلاج بدواء الالتهاب<br>Postpartum Infection and on kept antibiotic	☐ نزيف ما بعد الولادة<br>Prolonged haemorrhage

19. خلال الحمل الســابق هل حدث اي مضــاعفات صــحية متعلقة بالمولود؟

Did you face any previous new-born or foetal complication?

☐طفل مع اعاقة صحية

Baby with congenital anomaly

MRC-01-20-1129 Validity: 04 04 2020 - 12 07 2020 E-stamped 05 Apr 2021

☐لا يوجد لدي اي مشاكل صحية None

☐مشاكل صحية اخرى الرجاء التحديد… Others

دخول مستشفى ☐ Hospital admission

20. هل سـبق لك الاجهاض (وفاة الجنين حدث قبل الشـهر الخامس او قبل الاسـبوع العشـرين من الحمل)؟

Did you have any previous miscarriage or abortion (before your fifth month /<20week gestation)?

(1)Yes نعم☐ (0)No لا☐

21. هل سبق لك وفاة جنين بعد الشهر الخامس او بعد العشرين اسبوع من الحمل ؟

Did you experience still birth? (death of the fetus after twenty-week gestation).

Yes نعم (1) ☐ Noلا (0) ☐

ح-الأمراض المزمنة (قبل الحمل)

D-Medical history (illness before pregnancy-Chronic)

22. **هل تم تشـخيصـك بأي من الامراض المزمنة قبل الحمل الحالي(اكثر من ثلاثة اشهر)؟**

Have been diagnosed with any chronic medical conditions (more than3 month)?

Yes نعم☐ No لا☐

32. **ما هو المرض المزمن؟**

State the chronic condition:

☐مرض السكري من النوع الثاني Diabetes type II

Thyroid problem/مشكلة الغدة الدرقية☐

Anaemia/.فقر الدم☐

Kidney diseasesأمراض الكلى☐

Asthma/الربو☐

………..

☐ ارتفاع ضغط الدم High blood pressure/

☐امراض قلب Heart problems

☐ لم يتم تشخيصي باي من هذه الامراض None

☐ Others / امراض أخرى الرجاء التحدي..............................

ر -الأدوية التي تتناوليها حاليا اثناء فترة الحمل

G- Current Medications History

1) ما هي الأدوية او الفيتامينات التي تتناوليها خلال فترة الحمل ؟

In your current pregnancy: What medications or vitamins are you taking?

☐حمض الفولك Folic acid

☐الفيتامينات.......... Multivitamins are ….

☐ابر انسلين Insulin injections….

☐ملحق الغدة الدرقية Thyroid supplement

☐دواء مضاد للحموضة Antacid

☐أعشاب للاسترخاء الرجاء التحديد Relaxing Herbs /…...

☐دواء للسكري مثل جلوكوفيج Hypoglycaemic medication.

☐ Other mental illness related medication ادوية اخرى..........

☐ Antidepressant دواء للاكتئاب

☐لا اخذ اي دواء/None

الصحة النفسية-

-Mental health

2) هل تم تشخيصك باي مرض نفسي ؟

Have you been diagnosed with any mental illnesss?

☐القلق Anxiety

☐الاكتئاب Depression before pregnancy

☐نوبات الهلع Panic attacks

☐انفصام فى الشخصية. Schizophrenia

☐مرض ثنائي القطب Bipolar disease

☐لا لم يتم تشخيصي باي من هذه الامراض النفسية Others….

اكتئاب بعد الولادة Postpartum Depression ☐

Institutional Review Board Hamad Medical Corporation - Doha - Qatar Hamad

MRC-01-20-1129 Validity: 04 04 2020 - 12 07 2020 E-stamped 05 Apr 2021

3) هل اخذتي اي دواء مرتبط بالصحة النفسية اكثر من ستة مرات خلال اي سنة؟

Did you use to take any medications related to mental health more than 6 times in any year (Drugs like Valium, Xanax, Ativan, Klonopin, Ambien, Sonata, or Lunesta)?

No لا☐ Yes نعم☐

4) هل تلقيت أي علاج سلوكي معرفي؟

Did you receive any talk therapy - Cognitive Behavioral therapy (CBT) related to mental illness?

No لا☐ Yes نعم☐

5) هل لدي أسرتك (مثل الأجداد والأم او الاب والأشقاء) اي مرض نفسي؟

Do you have any mental illness running in your immediate family (e.g. grandparents, parents, siblings)?

No/كلا☐ I don’t know/لا اعلم ☐ Yes/نعم☐

س –عوامل نمط الحياة الحالية

H-Life style habits

6) هل تمارسين الرياضة المنتظمة (باستثناء العمل المنزلي)؟

Do you do regular sport different from your house activity?

No لا☐ Yes نعم☐

7) كم من الوقت تقضيه في الأنشطة الرياضية؟

How much time you spend on sport activities?

>20min اكثر من عشرين دقيقة☐ <20 min اقل من عشرين دقيقة☐

8) كم مرة في الأسبوع تقومين بالرياضة؟

How many times per week do you do sport?

اكثر من مرتين في الأسبوع☐ > 2 time/week

مرتين في الأسبوع☐ 2 time/week

اقل من مرة في الأسبوع☐ < 1 time/week.

MRC-01-20-1129 Validity: 04 04 2020 - 12 07 2020 E-stamped 05 Apr 2021

Fitness Score

9) <u>مؤشر النشاط البدني:</u>

☐ المؤشر صفر: غير لائق بدنيا: القيام بنشاط بدني أقل من مرة أو مرتين في الأسبوع ولمدة زمنية تقل عن 20 دقيقة.
☐ المؤشر واحد: نشيط :أ داء النشاط البدني مرة واحدة إلى مرتين في الأسبوع لمدة 20 دقيقة ، أو أكثر من مرتين في الأسبوع لمدة أقل من 20 دقيقة
☐ المؤشر اثنين : لائق بدنيا أداء النشاط البدني أكثر من مرتين في الأسبوع لأكثر من 20 دقيقة

- ☐ *<u>Not fit</u>*: perform physical activity less than once or twice a week and for a period of less than 20 minutes (zero index score)
- ☐ *<u>Active</u>*: perform physical activity once to twice a week for 20 minutes, or more than twice a week for less than 20 minutes have a physical activity index of one
- ☐ *<u>Fit</u>*: perform physical activity more than twice a week for more than 20 minutes

10) <u>هل تدخنين (الارغيلة او السيجار)؟</u>

Do you smoke? (Hubble-bubble, tobacco)

☐لم ادخن أبدًا

Never smoked

☐ادخن كل يوم

Every day smoker

☐ادخن حاليا ولكن ليس كل يوم

Current smoker but not every day

☐لا ادخن حالياً ولكن كنت ادخن سابقا (على الاقل 100 سيجارة في حياتي)

Former smoker (smoked at least 100 cigarettes but currently not smoking)

Institutional Review Board Hamad Medical Corporation - Doha - Qatar Hamad حمد

MRC-01-20-1129 Validity: 04 04 2020 - 12 07 2020 E-stamped 05 Apr 2021

Do you drink alcohol?

(11 هل تشربين الخمر؟

☐نعم اشرب حاليا خلال الحمل<br>Yes, I am drinking alcohol during my pregnancy.	☐حاليا لا اشرب ولكن كنت اشرب سابقا قبل الحمل<br>I don't drink now but I used to drink before pregnancy.	☐لا ابدا<br>No, never had alcohol before.

I- Stressful life events **ش –أحداث الحياة المجهدة**

Do you consider yourself living stressful life event?

(12 هل تواجهين أي حدث مرهق او مجهد او ازمة في حياتك حاليا؟

☐لا
No

☐نعم
Yes

If yes, kindly mention your stressful life event:

(13 اذا كان الجواب نعم ما هي الازمة؟...................

To what extent have you been stressed about specific baby gender?

(14 الى اي حد انت متوترة بخصوص جنس المولود؟

☐لا على الإطلاق	☐إلى حد صغير	☐بعض الاحيان	☐إلى حد كبير
Not at All	Very Little	Somewhat	To a Great Extent

How often do you face financial distress?

23. الى أي حد تواجهين ضائقة مالية؟

☐دائما	☐ أحيانا	☐ نادرًا	☐أبدا
Always (3)	Sometimes (2)	(1) Rarely	Never (0)

Institutional Review Board Hamad Medical Corporation - Doha - Qatar Hamad

MRC-01-20-1129 Validity: 04 04 2020 - 12 07 2020 E-stamped 05 Apr 2021

القسم الثاني

Section II: Standard Tools

Tool (1): Perinatal depression screening tool (EPDS)/اكتئاب الحمل

نرجو ان تختاري الجواب الأنسب الذي يعبر عن مشاعرك خلال الايام السبعة الماضية وليس مشاعرك اليوم فحسب (جواب واحد فقط) :

Please choose which comes closest to how you have felt IN THE PAST 7 DAYS, not just how you feel today (Only choose one answer):

Here is an example already completed — اليك مثل و قد اكمل:

I have felt Happy: — لقد شعرت بانني سعيدة:

☐كلا مطلقا<br>(3) Not at all	☐ كلا ليس في احوال كثيرة<br>(2) No, Not very often	☒ نعم معظم الاوقات<br>(1) Yes, some of the time	☐ نعم كل الاوقات<br>(0) Yes, most of the time

This would mean that: I have felt happy some of the time during the past week not just how you feel today. Please complete other questions in the same way:

و هذايعني لقد شعرت بانني سعيدة معظم الوقت خلال الاسبوع الماضي وليس مشاعرك اليوم فحسب

الرجاء ان تكملي الاسالة الاخرى بالطريقة ذاتها

In the past 7 days: — خلال الايام السبعة الماضية:

1. لقد تمكنت من الضحك و رؤية الجانب المضحك من الأمور

I have been able to laugh and see the funny side of things as much as I always could

☐كلا مطلقا<br>(3) Not at all	☐قطعا ليس بالمقدار نفسه الان<br>(2) Definitely not so much now	☐ليس تماما بالمقدار نفسه الان<br>(1) Not quite so much now.	☐بالمقدار الذي استطعته دائما<br>(0) As much as I always could

2. لقد تطلعت الى الامور بمتعة

I have looked forward with enjoyment to things

☐نادرا<br>(3) Hardly at all	☐قطعا اقل مما اعتدته<br>(2) Definitely less than I used to	☐نوع ما اقل مما اعتدته<br>(1) Rather less than I used to	☐بالمقدار نفسه مثل اي وقت مضى<br>(0) As much as I ever did

MRC-01-20-1129 Validity: 04 04 2020 - 12 07 2020 E-stamped 05 Apr 2021

3. لقد لمت نفسي بدون لزوم عندما سارت الامور على غير ما يرام*

I have blamed myself unnecessarily when things went wrong. *

☐كلا ابدا<br>(0) No, never	☐ليس اغلب الاحيان<br>(1) Not very often	☐نعم في بعض الاحيان<br>(2) Yes, some of the time	☐نعم في معظم الاحيان<br>(3) Yes, most of the time

4. لقد كنت قلقة ومشغولة البال من دون سبب وجيه

I have been anxious or worried for no good reasons

☐نعم اغلب الأحيان<br>(3) Yes, very often	☐نعم في بعض الاحيان<br>(2) Yes, sometimes	☐نادرا<br>(1) Hardly, ever	☐كلا مطلقا<br>(0) No, not at all

5. لقد شعرت بالخوف والذعر من دون سبب وجيه*

I have felt scared or panicky for no very good reason*

☐كلا مطلقا	☐كلا ليس كثيرا	☐نعم في بعض الاحيان	☐نعم اكتر الاحيان
(0) No, not at all	(1) No, not much	(2) Yes, sometimes	(3) Yes, quite a lot

6. تراكمت الاعمال علي فلم استطع القيام بها*

Things have been getting on top of me*

☐نعم في بعض الاحيان لم استطع القيام بها كالمعتاد<br>(2) Yes, sometimes I haven't been coping as well as usual	☐نعم في معظم الاحيان لم استطع القيام بها مطلقا<br>(3) Yes, most of the time I haven't been able to cope at all
☐كلا لقد استطعت القيام بها كالمعتاد<br>(0) No, I have been coping as well as ever	☐كلا لقد استطعت القيام بها في معظم الاحيان<br>(1) No, most of the time I have coped quite will

7. لقد كنت غير سعيدة لدرجة ان لدي صعوبة في النوم*

I have been so unhappy that I have had difficulty sleeping*

☐كلا مطلقا	☐ليس اغلب الاحيان	☐نعم في بعض الاحيان	☐نعم في معظم الاحيان

Institutional Review Board Hamad Medical Corporation · Doha · Qatar

MRC-01-20-1129 Validity: 04 04 2020 - 12 07 2020 E-stamped 05 Apr 2021

(0) No, not at all	(1) Not very often	(2) Yes, sometimes	(3) Yes, most of the time

I have felt sad or miserable*
.8 شعرت بانني حزينة وبائسة*

☐كلا مطلقا	☐كلا ليس اغلب الاحيان	☐نعم في اغلب الاحيان	☐نعم في معظم الاحيان
(0) No, not at all	(1) Not very often	(2) Yes, quite often	(3) Yes, most of the time

I have been so unhappy that I have been crying*
.9 لقد كنت غير سعيدة للغاية لدرجة اني كنت ابكي*

☐كلا ابدا<br>(0) No, not at all	☐فقط من وقت لأخر<br>(1) Only occasionally	☐نعم في اغلب الاحيان<br>(2) Yes, quite often	☐نعم في معظم الاحيان<br>(3) Yes, most of the time

The thought of harming myself has occurred to me*
.10 لقد خطرت لي فكرة الحاق الاذى بنفسي*

☐كلا ابدا	☐نادرا	☐نعم في بعض الاحيان	☐نعم في احوال كثيرة
(0) Never	(1) Hardly ever	(2) Sometimes	(3) Yes, quite often

Total Score of EPDS…

المجموع: ....

Research Questions Related to COVID-19 Phobia	“strongly disagree,”<br>لا أوافق بشدة	“disagree,”<br>لا أوافق	“neither agree nor disagree,”<br>لا اعلم	“agree<br>أوافق,”	“strongly agree<br>أوافق بشدة
1. I am most afraid of coronavirus-19.<br>أخشى أكثر من فيروس التاجي -19.	0	1	2	3	4

Institutional Review Board Hamad Medical Corporation · Doha · Qatar Hamad
MRC-01-20-1129 Validity: 04 04 2020 - 12 07 2020 E-stamped 05 Apr 2021

2.It makes me uncomfortable to think about coronavirus-19.<br><br>أشعر بعدم الارتياح للتفكير في فيروس كورونا -19	0	1	2	3	4
3.My hands become clammy when I think about coronavirus-19.<br><br>أصبحت يدي رطبة عندما أفكر في فيروس التاجي -19.	0	1	2	3	4
4.I am afraid of losing my life because of coronavirus-19.<br><br>أخشى أن أفقد حياتي بسبب فيروس كورونا -19.	0	1	2	3	4
5.When watching news and stories about coronavirus-19 on social media, I become nervous or anxious.<br><br>عندما أشاهد الأخبار والقصص حول فيروس التاجي -19 على وسائل التواصل الاجتماعي ، أصبت بالتوتر أو القلق.	0	1	2	3	4
6.I cannot sleep because I'm worrying about getting coronavirus-19.<br><br>لا أستطيع النوم لأنني قلق بشأن الإصابة بالفيروس التاجي -19	0	1	2	3	4
7.My heart races or palpitates when I think about getting coronavirus-19.<br><br>يسرع قلبي أو يخفق عندما أفكر في الإصابة بالفيروس التاجي -19.	0	1	2	3	4

Institutional Review Board Hamad Medical Corporation · Doha · Qatar

MRC-01-20-1129 Validity: 04 04 2020 - 12 07 2020 E-stamped 05 Apr 2021

Structured Questionnaire for T1 *(the participant is pregnant -14 to 42 weeks)*

Appendix IV

Structured Questionnaire- Video telecommunication

تاريخ جمع البيانات	عدم المشاركة	اسم جامع البيانات	رقم المشاركة
Date	Drop out	Data Collector Name	Participant Number
………/……. / …..	....	....	....

((بما انك حامل وصحتك تهمنا نتمنى ان ناخذ من وقتك خمسة عشر دقيقة لنسالك بعض الاسئلة .القسم الأول سيشمل مقابلة بحيث يسالك الباحث بعض الاسئلة. اما القسم الثاني فتقرير ذاتي تمليه المشتركة .))

As you are pregnant and your health status matters to us, we would like to ask you some questions. It will include face-to-face interview-based questionnaire and standard tools through the video telecommunication. It will not take more than 15 minutes.

Section I: Questionnaire / الجزء الاول: الاستبيان

يستند هذا الاستبيان على مقابلة ستسالين عن تاريخك الطبي وعادات أسلوب حياتك ودعم زوجك والمساعدة التي تتوقعينها بعد الولادة.

This is an interview-based questionnaire, going to investigate some aspects related to medical history and life style habit. Additionally, you will be asked about your partner and the expected postpartum support .

MRC-01-20-1129 Validity: 04 04 2020 - 12 07 2020 E-stamped 05 Apr 2021

1. هل تشتكين من اي عوارض صحية؟ Do you have any health complain?
كلا/(0)No- نعم /Yes (1)

- ☐ ارهاق/Fatigue
- ☐ الم في الظهر/Back pain
- ☐ تقيىء شديد/Vomiting
- ☐ صداع الراس /Headache
- ☐ اضطراب النوم /Sleep disturbance
- ☐ Others

2. خلال حملك الحالي هل تم تشخيصك بأي من الامراض الصحية المرتبطة بالحمل ؟
In your current pregnancy, have you been diagnosed with any pregnancy related health Problem/s?

- ☐ Hyperemesis Gravidarium تقيىء شديد
- ☐ Gestational Diabetes/ سكر الحمل
- ☐ ....Abnormal foetal سونار الجنين غير طبيعي ultrasound
- ☐ مشاكل صحية اخرى..................................
- ☐ High blood pressure related to pregnancy/ضغط مرتفع مرتبط بالحمل
- ☐ Anaemia in pregnancy فقر الدم في الحمل
  Less than 3month/ اقل من ثلاث اشهر
- Suspected Corona ☐
- Confirmed Corona ☐

س –عوامل نمط الحياة الحالية

H-Life style habits

(15 هل تمارسين الرياضة المنتظمة (باستثناء العمل المنزلي)؟
Do you do regular sport different from your house activity?

☐نعم Yes ☐لا No

(16 كم من الوقت تقضيه في الأنشطة الرياضية؟
How much time you spend on sport activities?

☐اقل من عشرين دقيقة <20 min ☐اكثر من عشرين دقيقة >20min

Institutional Review Board Hamad Medical Corporation · Doha · Qatar
MRC-01-20-1129 Validity: 04 04 2020 - 12 07 2020 E-stamped 05 Apr 2021

(17 كم مرة في الأسبوع تقومين بالرياضة؟

How many times per week do you do sport?

☐اقل من مرة في الأسبوع<br>< 1 time/week.	☐مرتين في الأسبوع<br>2 time/week	☐اكثر من مرتين في الأسبوع<br>> 2 time/week

(18 مؤشر النشاط البدني:

Fitness Score

- ☐ المؤشر صفر: غير لائق بدنيا: القيام بنشاط بدني أقل من مرة أو مرتين في الأسبوع ولمدة زمنية تقل عن 20 دقيقة.
- ☐ المؤشر واحد: نشيط :أ داء النشاط البدني مرة واحدة إلى مرتين في الأسبوع لمدة 20 دقيقة ، أو أكثر من مرتين في الأسبوع لمدة أقل من 20 دقيقة
- ☐ المؤشر اثنين : لائق بدنيا أداء النشاط البدني أكثر من مرتين في الأسبوع لأكثر من 20 دقيقة

- ☐ *Not fit*: perform physical activity less than once or twice a week and for a period of less than 20 minutes (zero index score)
- ☐ *Active*: perform physical activity once to twice a week for 20 minutes, or more than twice a week for less than 20 minutes have a physical activity index of one
- ☐ *Fit*: perform physical activity more than twice a week for more than 20 minutes

(19 هل تدخنين (الارغيلة او السيجار)؟

Do you smoke? (Hubble-bubble, tobacco)

☐لم ادخن أبدًا<br>Never smoked	☐لا ادخن حالياً ولكن كنت ادخن سابقا (على الاقل 100 سيجارة في حياتي)<br>Former smoker (smoked at least 100 cigarettes but currently not smoking)	☐ادخن حاليا ولكن ليس كل يوم<br>Current smoker but not every day	☐ادخن كل يوم<br>Every day smoker

Institutional Review Board Hamad Medical Corporation - Doha - Qatar

MRC-01-20-1129 Validity: 04 04 2020 - 12 07 2020 E-stamped 05 Apr 2021

20) هل تشربين الخمر؟

Do you drink alcohol?

☐نعم اشرب حاليا خلال الحمل<br>Yes, I am drinking alcohol during my pregnancy.	☐حاليا لا اشرب ولكن كنت اشرب سابقا قبل الحمل<br>I don't drink now but I used to drink before pregnancy.	☐لا ابدا<br>No, never had alcohol before.

I- Stressful life events | **ش –أحداث الحياة المجهدة**

21) هل تواجهين أي حدث مرهق او مجهد او ازمة في حياتك حاليا؟

Do you consider yourself living stressful life event?

☐لا<br>No	☐نعم<br>Yes

22) اذا كان الجواب نعم ما هي الازمة؟......................

If yes, kindly mention your stressful life event:

23) الى اي حد انت متوترة بخصوص جنس المولود؟

To what extent have you been stressed about specific baby gender?

☐لا على الإطلاق<br>Not at All	☐إلى حد صغير<br>Very Little	☐بعض الاحيان<br>Somewhat	☐إلى حد كبير<br>To a Great Extent

---

24. الى أي حد تواجهين ضائقة مالية؟

How often do you face financial distress?

☐دائما<br>Always (3)	☐ أحيانا<br>Sometimes (2)	☐ نادرًا<br>(1) Rarely	☐أبدا<br>Never (0)

MRC-01-20-1129 Validity: 04 04 2020 - 12 07 2020 E-stamped 05 Apr 2021

Section II: Standard Tools

Tool (1): Perinatal depression screening tool (EPDS)/اكتئاب الحمل

نرجو ان تختاري الجواب الأنسب الذي يعبر عن مشاعرك خلال الايام السبعة الماضية وليس مشاعرك اليوم فحسب (جواب واحد فقط) :

Please choose which comes closest to how you have felt IN THE PAST 7 DAYS, not just how you feel today (Only choose one answer):

Here is an example already completed — اليك مثل و قد اكمل:

I have felt Happy: — لقد شعرت بانني سعيدة:

☐كلا مطلقا<br>(3) Not at all	☐ كلا ليس في احوال كثيرة<br>(2) No, Not very often	☒ نعم معظم الاوقات<br>(1) Yes, some of the time	☐ نعم كل الاوقات<br>(0) Yes, most of the time

This would mean that: I have felt happy some of the time during the past week not just how you feel today. Please complete other questions in the same way:

و هذايعني لقد شعرت بانني سعيدة معظم الوقت خلال الاسبوع الماضي وليس مشاعرك اليوم فحسب

الرجاء ان تكملي الاسالة الاخرى بالطريقة ذاتها

In the past 7 days: — خلال الايام السبعة الماضية:

11. لقد تمكنت من الضحك و رؤية الجانب المضحك من الأمور

I have been able to laugh and see the funny side of things as much as I always could

☐كلا مطلقا<br>(3) Not at all	☐قطعا ليس بالمقدار نفسه الان<br>(2) Definitely not so much now	☐ليس تماما بالمقدار نفسه الان<br>(1) Not quite so much now.	☐بالمقدار الذي استطعته دائما<br>(0) As much as I always could

12. لقد تطلعت الى الامور بمتعة

I have looked forward with enjoyment to things

☐نادرا<br>(3) Hardly at all	☐قطعا اقل مما اعتدته<br>(2) Definitely less than I used to	☐نوع ما اقل مما اعتدته<br>(1) Rather less than I used to	☐بالمقدار نفسه مثل اي وقت مضى<br>(1) As much as I ever did

Institutional Review Board · Hamad Medical Corporation · Doha · Qatar

MRC-01-20-1129 Validity: 04 04 2020 - 12 07 2020 E-stamped 05 Apr 2021

13.لقد لمت نفسي بدون لزوم عندما سارت الامور على غير ما يرام*

I have blamed myself unnecessarily when things went wrong. *

☐كلا ابدا<br>(0) No, never	☐ليس اغلب الاحيان<br>(1) Not very often	☐نعم في بعض الاحيان<br>(2) Yes, some of the time	☐نعم في معظم الاحيان<br>(3) Yes, most of the time

14.لقد كنت قلقة ومشغولة البال من دون سبب وجيه

I have been anxious or worried for no good reasons

☐نعم اغلب الأحيان<br>(3) Yes, very often	☐نعم في بعض الاحيان<br>(2) Yes, sometimes	☐نادرا<br>(1) Hardly, ever	☐كلا مطلقا<br>(0) No, not at all

15. لقد شعرت بالخوف والذعر من دون سبب وجيه*

I have felt scared or panicky for no very good reason*

☐كلا مطلقا	☐كلا ليس كثيرا	☐نعم في بعض الاحيان	☐نعم اكثر الاحيان
(0) No, not at all	(1) No, not much	(2) Yes, sometimes	(3) Yes, quite a lot

16. تراكمت الاعمال علي فلم استطع القيام بها*

Things have been getting on top of me*

☐نعم في بعض الاحيان لم استطع القيام بها كالمعتاد<br>(2) Yes, sometimes I haven't been coping as well as usual	☐نعم في معظم الاحيان لم استطع القيام بها مطلقا<br>(3) Yes, most of the time I haven't been able to cope at all
☐كلا لقد استطعت القيام بها كالمعتاد<br>(0) No, I have been coping as well as ever	☐كلا لقد استطعت القيام بها في معظم الاحيان<br>(1) No, most of the time I have coped quite will

17. لقد كنت غير سعيدة لدرجة ان لدي صعوبة في النوم*

I have been so unhappy that I have had difficulty sleeping*

☐كلا مطلقا<br>(0) No, not at all	☐ليس اغلب الاحيان<br>(1) Not very often	☐نعم في بعض الاحيان<br>(2) Yes, sometimes	☐نعم في معظم الاحيان<br>(3) Yes, most of the time

MRC-01-20-1129 Validity: 04 04 2020 - 12 07 2020 E-stamped 05 Apr 2021

I have felt sad or miserable*   18. شعرت بانني حزينة وبائسة*

☐كلا مطلقا	☐كلا ليس اغلب الاحيان	☐نعم في اغلب الاحيان	☐نعم في معظم الاحيان
(0) No, not at all	(1) Not very often	(2) Yes, quite often	(3) Yes, most of the time

I have been so unhappy that I have been crying*   19. لقد كنت غير سعيدة للغاية لدرجة اني كنت ابكي*

☐كلا ابدا<br>(0) No, not at all	☐فقط من وقت لأخر<br>(1) Only occasionally	☐نعم في اغلب الاحيان<br>(2) Yes, quite often	☐نعم في معظم الاحيان<br>(3) Yes, most of the time

The thought of harming myself has occurred to me*   20. لقد خطرت لي فكرة الحاق الاذى بنفسي*

☐كلا ابدا<br>(0) Never	☐نادرا<br>(1) Hardly ever	☐نعم في بعض الاحيان<br>(2) Sometimes	☐نعم في احوال كثيرة<br>(3) Yes, quite often

Total Score of EPDS…

المجموع: ....

Research Questions Related to COVID-19 Phobia	"strongly disagree,"<br>لا أوافق بشدة	"disagree,"<br>لا أوافق	"neither agree nor disagree,"<br>لا اعلم	"agree<br>أوافق,"	"strongly agree<br>أوافق بشدة
2. I am most afraid of coronavirus-19.<br>أخشى أكثر من فيروس التاجي 19-.	0	1	2	3	4
2.It makes me uncomfortable to think about coronavirus-19.	0	1	2	3	4

Institutional Review Board Hamad Medical Corporation · Doha · Qatar Hamad

MRC-01-20-1129 Validity: 04 04 2020 - 12 07 2020 E-stamped 05 Apr 2021

أشعر بعدم الارتياح للتفكير في فيروس كورونا -19					
3.My hands become clammy when I think about coronavirus-19.<br><br>أصبحت يدي رطبة عندما أفكر في فيروس التاجي -19.	0	1	2	3	4
4.I am afraid of losing my life because of coronavirus-19.<br><br>أخشى أن أفقد حياتي بسبب فيروس كورونا -19.	0	1	2	3	4
5.When watching news and stories about coronavirus-19 on social media, I become nervous or anxious.<br><br>عندما أشاهد الأخبار والقصص حول فيروس التاجي -19 على وسائل التواصل الاجتماعي ، أصبت بالتوتر أو القلق.	0	1	2	3	4
6.I cannot sleep because I'm worrying about getting coronavirus-19.<br><br>لا أستطيع النوم لأنني قلق بشأن الإصابة بالفيروس التاجي -19	0	1	2	3	4
7.My heart races or palpitates when I think about getting coronavirus-19.<br><br>يسرع قلبي أو يخفق عندما أفكر في الإصابة بالفيروس التاجي -19.	0	1	2	3	4

Institutional Review Board
Hamad
Hamad Medical Corporation - Doha - Qatar

MRC-01-20-1129 Validity: 04 04 2020 - 12 07 2020 E-stamped 05 Apr 2021

Structured Questionnaire for T2 to T5 *(the participant is postpartum)*

T2

Appendix III

Structured Questionnaire- Video telecommunication

**تاريخ جمع البيانات**	عدم المشاركة	**اسم جامع البيانات**	**رقم المشاركة**
Date	Drop out	Data Collector Name	Participant Number
………/……. /…..	....	....	....

((بما انك حامل وصحتك تهمنا نتمنى ان ناخذ من وقتك خمسة عشر دقيقة لنسالك بعض الاسئلة .القسم الأول سيشمل مقابلة بحيث يسالك الباحث بعض الاسئلة. اما القسم الثاني فتقرير ذاتي تمليه المشتركة .))

Congratulation on your baby! As you are in your postpartum period and your health status matters to us, we would like to ask you some questions. It will include face-to-face interview-based questionnaire and standard tools through the video telecommunication. It will not take more than 15 minutes.

Section I: Questionnaire / الجزء الاول: الاستبيان

يستند هذا الاستبيان على مقابلة تقوم بالتحقيق في بعض الجوانب المتعلقة بحياتك الاجتماعية والديموغرافية والاقتصادية. كما ستسالين عن تاريخك الطبي وعادات أسلوب حياتك ودعم زوجك والمساعدة التي تتوقعينها بعد الولادة.

This is an interview-based questionnaire, going to investigate some aspects related to socio demographical factors, economic factors, medical history and life style habit. Additionally, you will be asked about your partner and the expected postpartum support .

MRC-01-20-1129 Validity: 04 04 2020 - 12 07 2020 E-stamped 05 Apr 2021

1. Mother health: Any health complication……………
2. Baby health: any complication….

س –عوامل نمط الحياة الحالية

H-Life style habits

Do you do regular sport different from your house activity?

(24 هل تمارســـين الرياضـــة المنتظمة (باســـتثناء العمل المنزلي)؟

No لا☐ Yes نعم☐

(25 كم من الوقت تقضيه في الأنشطة الرياضية؟

How much time you spend on sport activities?

>20min اكثر من عشرين دقيقة☐ <20 min اقل من عشرين دقيقة☐

(26 كم مرة في الأسبوع تقومين بالرياضة؟

How many times per week do you do sport?

اكثر من مرتين في الأسبوع☐ > 2 time/week

مرتين في الأسبوع☐ 2 time/week

اقل من مرة في الأسبوع☐ < 1 time/week.

Fitness Score

(27 مؤشر النشاط البدني:

☐ المؤشر صفر: غير لائق بدنيا: القيام بنشاط بدني أقل من مرة أو مرتين في الأسبوع ولمدة زمنية تقل عن 20 دقيقة.
☐ المؤشر واحد: نشيط :أ داء النشاط البدني مرة واحدة إلى مرتين في الأسبوع لمدة 20 دقيقة ، أو أكثر من مرتين في الأسبوع لمدة أقل من 20 دقيقة
☐ المؤشر اثنين : لائق بدنيا أداء النشاط البدني أكثر من مرتين في الأسبوع لأكثر من 20 دقيقة

☐ *Not fit*: perform physical activity less than once or twice a week and for a period of less than 20 minutes (zero index score)
☐ *Active*: perform physical activity once to twice a week for 20 minutes, or more than twice a week for less than 20 minutes have a physical activity index of one
☐ *Fit*: perform physical activity more than twice a week for more than 20 minutes

Institutional Review Board Hamad Medical Corporation - Doha - Qatar

MRC-01-20-1129 Validity: 04 04 2020 - 12 07 2020 E-stamped 05 Apr 2021

Do you smoke? (Hubble-bubble, tobacco)

(28 هل تدخنين (الارغيلة او السيجار)؟

☐لم ادخن أبدًا

Never smoked

☐ادخن كل يوم

Every day smoker

☐ادخن حاليا ولكن ليس كل يوم

Current smoker but not every day

☐لا ادخن حالياً ولكن كنت ادخن سابقا (على الاقل 100 سيجارة في حياتي)

Former smoker (smoked at least 100 cigarettes but currently not smoking)

Do you drink alcohol?

(29 هل تشربين الخمر؟

☐نعم اشرب حاليا خلال الحمل

Yes, I am drinking alcohol during my pregnancy.

☐حاليا لا اشرب ولكن كنت اشرب سابقا قبل الحمل

I don't drink now but I used to drink before pregnancy.

☐لا ابدا

No, never had alcohol before.

I- Stressful life events

ش –أحداث الحياة المجهدة

Do you consider yourself living stressful life event?

(30 هل تواجهين أي حدث مرهق او مجهد او ازمة في حياتك حاليا؟

☐نعم

Yes

☐لا

No

If yes, kindly mention your stressful life event:

(31 اذا كان الجواب نعم ما هي الازمة؟.....................

(32 الى اي حد انت متوترة بخصوص جنس المولود؟

MRC-01-20-1129 Validity: 04 04 2020 - 12 07 2020 E-stamped 05 Apr 2021

To what extent have you been stressed about specific baby gender?

☐لا على الإطلاق<br>Not at All	☐إلى حد صغير<br>Very Little	☐بعض الاحيان<br>Somewhat	☐إلى حد كبير<br>To a Great Extent

25. الى أي حد تواجهين ضائقة مالية؟

How often do you face financial distress?

☐دائما<br>Always (3)	☐ أحيانا<br>Sometimes (2)	☐ نادرًا<br>(1) Rarely	☐أبدا<br>Never (0)

القسم الثاني

Section II: Standard Tools

Tool (1): Perinatal depression screening tool (EPDS)/اكتئاب الحمل

نرجو ان تختاري الجواب الأنسب الذي يعبر عن مشاعرك خلال الايام السبعة الماضية وليس مشاعرك اليوم فحسب (جواب واحد فقط) :

Please choose which comes closest to how you have felt IN THE PAST 7 DAYS, not just how you feel today (Only choose one answer):

Here is an example already completed

اليك مثل و قد اكمل:

I have felt Happy:

لقد شعرت بانني سعيدة:

☐كلا مطلقا<br>(3) Not at all	☐ كلا ليس في احوال كثيرة<br>(2) No, Not very often	☒ نعم معظم الاوقات<br>(1) Yes, some of the time	☐ نعم كل الاوقات<br>(0) Yes, most of the time

This would mean that: I have felt happy some of the time during the past week not just how you feel today. Please complete other questions in the same way:

و هذايعني لقد شعرت بانني سعيدة معظم الوقت خلال الاسبوع الماضي وليس مشاعرك اليوم فحسب

الرجاء ان تكملي الاسالة الاخرى بالطريقة ذاتها

In the past 7 days:

خلال الايام السبعة الماضية:

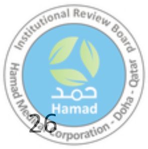

MRC-01-20-1129 Validity: 04 04 2020 - 12 07 2020 E-stamped 05 Apr 2021

I have been able to laugh and see the funny side of things as much as I always could

3. لقد تمكنت من الضحك و رؤية الجانب المضحك من الأمور

☐كلا مطلقا<br><br>(3) Not at all	☐قطعا ليس بالمقدار نفسه الان<br><br>(2) Definitely not so much now	☐ليس تماما بالمقدار نفسه الان<br><br>(1) Not quite so much now.	☐بالمقدار الذي استطعته دائما<br><br>(0) As much as I always could

I have looked forward with enjoyment to things

4. لقد تطلعت الى الامور بمتعة

☐نادرا<br><br>(3) Hardly at all	☐قطعا اقل مما اعتدته<br><br>(2) Definitely less than I used to	☐نوع ما اقل مما اعتدته<br><br>(1) Rather less than I used to	☐بالمقدار نفسه مثل اي وقت مضى<br><br>(2) As much as I ever did

I have blamed myself unnecessarily when things went wrong. *

5. لقد لمت نفسي بدون لزوم عندما سارت الامور على غير ما يرام*

☐كلا ابدا<br><br>(0) No, never	☐ليس اغلب الاحيان<br><br>(1) Not very often	☐نعم في بعض الاحيان<br><br>(2) Yes, some of the time	☐نعم في معظم الاحيان<br><br>(3) Yes, most of the time

I have been anxious or worried for no good reasons

6. لقد كنت قلقة ومشغولة البال من دون سبب وجيه

☐نعم اغلب الأحيان<br><br>(3) Yes, very often	☐نعم في بعض الاحيان<br><br>(2) Yes, sometimes	☐نادرا<br><br>(1) Hardly, ever	☐كلا مطلقا<br><br>(0) No, not at all

I have felt scared or panicky for no very good reason*

7. لقد شعرت بالخوف والذعر من دون سبب وجيه*

☐كلا مطلقا	☐كلا ليس كثيرا	☐نعم في بعض الاحيان	☐نعم اكثر الاحيان
(0) No, not at all	(1) No, not much	(2) Yes, sometimes	(3) Yes, quite a lot

Institutional Review Board
Hamad Medical Corporation - Doha - Qatar
حمد
Hamad

MRC-01-20-1129 Validity: 04 04 2020 - 12 07 2020 E-stamped 05 Apr 2021

8. تراكمت الاعمال علي فلم استطع القيام بها*

Things have been getting on top of me*

☐نعم في بعض الاحيان لم استطع القيام بها كالمعتاد<br>(2) Yes, sometimes I haven’t been coping as well as usual	☐نعم في معظم الاحيان لم استطع القيام بها مطلقا<br>(3) Yes, most of the time I haven’t been able to cope at all
☐كلا لقد استطعت القيام بها كالمعتاد<br>(0) No, I have been coping as well as ever	☐كلا لقد استطعت القيام بها في معظم الاحيان<br>(1) No, most of the time I have coped quite will

9. لقد كنت غير سـعيدة لدرجة ان لدي صـعوبة في النوم*

I have been so unhappy that I have had difficulty sleeping*

☐كلا مطلقا<br>(0) No, not at all	☐ليس اغلب الاحيان<br>(1) Not very often	☐نعم في بعض الاحيان<br>(2) Yes, sometimes	☐نعم في معظم الاحيان<br>(3) Yes, most of the time

10. شعرت بانني حزينة وبائسة*

I have felt sad or miserable*

☐كلا مطلقا	☐كلا ليس اغلب الاحيان	☐نعم في اغلب الاحيان	☐نعم في معظم الاحيان
(0) No, not at all	(1) Not very often	(2) Yes, quite often	(3) Yes, most of the time

11. لقد كنت غير سعيدة للغاية لدرجة اني كنت ابكي*

I have been so unhappy that I have been crying*

☐كلا ابدا<br>(0) No, not at all	☐فقط من وقت لأخر<br>(1) Only occasionally	☐نعم في اغلب الاحيان<br>(2) Yes, quite often	☐نعم في معظم الاحيان<br>(3) Yes, most of the time

12. لقد خطرت لي فكرة الحاق الاذى بنفسي*

The thought of harming myself has occurred to me*

☐كلا ابدا<br>(0) Never	☐نادرا<br>(1) Hardly ever	☐نعم في بعض الاحيان<br>(2) Sometimes	☐نعم في احوال كثيرة<br>(3) Yes, quite often

Institutional Review Board Hamad Medical Corporation · Doha · Qatar

MRC-01-20-1129 Validity: 04 04 2020 - 12 07 2020 E-stamped 05 Apr 2021

Total Score of EPDS…

المجموع: ....

Research Questions Related to COVID-19 Phobia	"strongly disagree," لا أوافق بشدة	"disagree," لا أوافق	"neither agree nor disagree," لا اعلم	"agree أوافق,"	"strongly agree أوافق بشدة
3. I am most afraid of coronavirus-19. أخشى أكثر من فيروس التاجي -19.	0	1	2	3	4
2.It makes me uncomfortable to think about coronavirus-19. أشعر بعدم الارتياح للتفكير في فيروس كورونا -19	0	1	2	3	4
3.My hands become clammy when I think about coronavirus-19. أصبحت يدي رطبة عندما أفكر في فيروس التاجي -19.	0	1	2	3	4
4.I am afraid of losing my life because of coronavirus-19. أخشى أن أفقد حياتي بسبب فيروس كورونا -19.	0	1	2	3	4
5.When watching news and stories about coronavirus-19 on social media, I become nervous or anxious. عندما أشاهد الأخبار والقصص حول فيروس التاجي -19 على وسائل التواصل الاجتماعي ، أصبت بالتوتر أو القلق.	0	1	2	3	4
6.I cannot sleep because I'm worrying about getting coronavirus-19.	0	1	2	3	4

Institutional Review Board Hamad Medical Corporation · Doha · Qatar Hamad

MRC-01-20-1129 Validity: 04 04 2020 - 12 07 2020 E-stamped 05 Apr 2021

لا أستطيع النوم لأنني قلق بشأن الإصابة بالفيروس التاجي -19					
7.My heart races or palpitates when I think about getting coronavirus-19.<br><br>يسرع قلبي أو يخفق عندما أفكر في الإصابة بالفيروس التاجي -19.	0	1	2	3	4

Institutional Review Board
Hamad Medical Corporation - Doha - Qatar
Hamad

MRC-01-20-1129 Validity: 04 04 2020 - 12 07 2020 E-stamped 05 Apr 2021

Structured Questionnaire for T2 to T5 *(the participant is postpartum)*

Circle the number of session:

T3

Appendix III

Structured Questionnaire- Video telecommunication

تاريخ جمع البيانات	عدم المشاركة	اسم جامع البيانات	رقم المشاركة
Date	Drop out	Data Collector Name	Participant Number
………/……. / ……	....	....	....

((بما انك حامل وصحتك تهمنا نتمنى ان ناخذ من وقتك خمسة عشر دقيقة لنسالك بعض الاسئلة .القسم الأول سيشمل مقابلة بحيث يسالك الباحث بعض الاسئلة. اما القسم الثاني فتقرير ذاتي تمليه المشتركة .))

Congratulation on your baby! As you are in your postpartum period and your health status matters to us, we would like to ask you some questions. It will include face-to-face interview-based questionnaire and standard tools through the video telecommunication. It will not take more than 15 minutes.

Section I: Questionnaire / الجزء الاول: الاستبيان

يستند هذا الاستبيان على مقابلة تقوم بالتحقيق في بعض الجوانب المتعلقة بحياتك الاجتماعية والديموغرافية والاقتصادية. كما ستسالين عن تاريخك الطبي وعادات أسلوب حياتك ودعم زوجك والمساعدة التي تتوقعينها بعد الولادة.

This is an interview-based questionnaire, going to investigate some aspects related to socio demographical factors, economic factors, medical history and life style habit. Additionally, you will be asked about your partner and the expected postpartum support .

MRC-01-20-1129 Validity: 04 04 2020 - 12 07 2020 E-stamped 05 Apr 2021

13. Mother health: Any health complication…………..
14. Baby health: any complication….

س –عوامل نمط الحياة الحالية

H-Life style habits

Do you do regular sport different from your house activity?

(33 هل تمارســين الرياضــة المنتظمة (باســتثناء العمل المنزلي)؟

No لا☐ Yes نعم☐

How much time you spend on sport activities?

(34 كم من الوقت تقضيه في الأنشطة الرياضية؟

>20min اكثر من عشرين دقيقة☐ <20 min اقل من عشرين دقيقة☐

How many times per week do you do sport?

(35 كم مرة في الأسبوع تقومين بالرياضة؟

اكثر من مرتين في الأسبوع☐ > 2 time/week

مرتين في الأسبوع☐ 2 time/week

اقل من مرة في الأسبوع☐ < 1 time/week.

Fitness Score

(36 مؤشر النشاط البدني:

☐ المؤشر صفر: غير لائق بدنيا: القيام بنشاط بدني أقل من مرة أو مرتين في الأسبوع ولمدة زمنية تقل عن 20 دقيقة.
☐ المؤشر واحد: نشيط :أ داء النشاط البدني مرة واحدة إلى مرتين في الأسبوع لمدة 20 دقيقة ، أو أكثر من مرتين في الأسبوع لمدة أقل من 20 دقيقة
☐ المؤشر اثنين : لائق بدنيا أداء النشاط البدني أكثر من مرتين في الأسبوع لأكثر من 20 دقيقة

☐ *Not fit*: perform physical activity less than once or twice a week and for a period of less than 20 minutes (zero index score)
☐ *Active*: perform physical activity once to twice a week for 20 minutes, or more than twice a week for less than 20 minutes have a physical activity index of one
☐ *Fit*: perform physical activity more than twice a week for more than 20 minutes

Institutional Review Board Hamad Medical Corporation · Doha · Qatar

MRC-01-20-1129 Validity: 04 04 2020 - 12 07 2020 E-stamped 05 Apr 2021

(37 هل تدخنين (الارغيلة او السيجار)؟

Do you smoke? (Hubble-bubble, tobacco)

☐لم ادخن أبدًا

Never smoked

☐لا ادخن حالياً ولكن كنت ادخن سابقا (على الاقل 100 سيجارة في حياتي)

Former smoker (smoked at least 100 cigarettes but currently not smoking)

☐ادخن حاليا ولكن ليس كل يوم

Current smoker but not every day

☐ادخن كل يوم

Every day smoker

(38 هل تشربين الخمر؟

Do you drink alcohol?

☐لا ابدا

No, never had alcohol before.

☐حاليا لا اشرب ولكن كنت اشرب سابقا قبل الحمل

I don't drink now but I used to drink before pregnancy.

☐نعم اشرب حاليا خلال الحمل

Yes, I am drinking alcohol during my pregnancy.

I- Stressful life events

ش –أحداث الحياة المجهدة

(39 هل تواجهين أي حدث مرهق او مجهد او ازمة في حياتك حاليا؟

Do you consider yourself living stressful life event?

☐نعم

Yes

☐لا

No

(40 اذا كان الجواب نعم ما هي الازمة؟....................

If yes, kindly mention your stressful life event:

(41 الى اي حد انت متوترة بخصوص جنس المولود؟

Institutional Review Board Hamad Medical Corporation - Doha - Qatar حمد Hamad

MRC-01-20-1129 Validity: 04 04 2020 - 12 07 2020 E-stamped 05 Apr 2021

To what extent have you been stressed about specific baby gender?

☐لا على الإطلاق<br>Not at All	☐إلى حد صغير<br>Very Little	☐بعض الاحيان<br>Somewhat	☐إلى حد كبير<br>To a Great Extent

---

26. الى أي حد تواجهين ضائقة مالية؟

How often do you face financial distress?

☐دائما<br>Always (3)	☐ أحيانا<br>Sometimes (2)	☐ نادرًا<br>(1) Rarely	☐أبدا<br>Never (0)

القسم الثاني

Section II: Standard Tools

Tool (1): Perinatal depression screening tool (EPDS)/اكتئاب الحمل

نرجو ان تختاري الجواب الأنسب الذي يعبر عن مشاعرك خلال الايام السبعة الماضية وليس مشاعرك اليوم فحسب (جواب واحد فقط) :

Please choose which comes closest to how you have felt IN THE PAST 7 DAYS, not just how you feel today (Only choose one answer):

Here is an example already completed

اليك مثل و قد اكمل:

I have felt Happy:

لقد شعرت بانني سعيدة:

☐كلا مطلقا<br><br>(3) Not at all	☐ كلا ليس في احوال كثيرة<br><br>(2) No, Not very often	☒ نعم معظم الاوقات<br><br>(1) Yes, some of the time	☐ نعم كل الاوقات<br><br>(0) Yes, most of the time

This would mean that: I have felt happy some of the time during the past week not just how you feel today. Please complete other questions in the same way:

و هذايعني لقد شعرت بانني سعيدة معظم الوقت خلال الاسبوع الماضي وليس مشاعرك اليوم فحسب

الرجاء ان تكملي الاسالة الاخرى بالطريقة ذاتها

In the past 7 days:

خلال الايام السبعة الماضية:

MRC-01-20-1129 Validity: 04 04 2020 - 12 07 2020 E-stamped 05 Apr 2021

15. لقد تمكنت من الضحك و رؤية الجانب المضحك من الأمور

I have been able to laugh and see the funny side of things as much as I always could

☐كلا مطلقا<br>(3) Not at all	☐قطعا ليس بالمقدار نفسه الان<br>(2) Definitely not so much now	☐ليس تماما بالمقدار نفسه الان<br>(1) Not quite so much now.	☐بالمقدار الذي استطعته دائما<br>(0) As much as I always could

16. لقد تطلعت الى الامور بمتعة

I have looked forward with enjoyment to things

☐نادرا<br>(3) Hardly at all	☐قطعا اقل مما اعتدته<br>(2) Definitely less than I used to	☐نوع ما اقل مما اعتدته<br>(1) Rather less than I used to	☐بالمقدار نفسه مثل اي وقت مضى<br>(3) As much as I ever did

17. لقد لمت نفسي بدون لزوم عندما سارت الامور على غير ما يرام*

I have blamed myself unnecessarily when things went wrong. *

☐كلا ابدا<br>(0) No, never	☐ليس اغلب الاحيان<br>(1) Not very often	☐نعم في بعض الاحيان<br>(2) Yes, some of the time	☐نعم في معظم الاحيان<br>(3) Yes, most of the time

18. لقد كنت قلقة ومشغولة البال من دون سبب وجيه

I have been anxious or worried for no good reasons

☐نعم اغلب الأحيان<br>(3) Yes, very often	☐نعم في بعض الاحيان<br>(2) Yes, sometimes	☐نادرا<br>(1) Hardly, ever	☐كلا مطلقا<br>(0) No, not at all

19. لقد شعرت بالخوف والذعر من دون سبب وجيه*

I have felt scared or panicky for no very good reason*

☐كلا مطلقا	☐كلا ليس كثيرا	☐نعم في بعض الاحيان	☐نعم اكثر الاحيان
(0) No, not at all	(1) No, not much	(2) Yes, sometimes	(3) Yes, quite a lot

MRC-01-20-1129 Validity: 04 04 2020 - 12 07 2020 E-stamped 05 Apr 2021

20. تراكمت الاعمال علي فلم استطع القيام بها*

Things have been getting on top of me*

☐نعم في بعض الاحيان لم استطع القيام بها كالمعتاد<br>(2) Yes, sometimes I haven't been coping as well as usual	☐نعم في معظم الاحيان لم استطع القيام بها مطلقا<br>(3) Yes, most of the time I haven't been able to cope at all
☐كلا لقد استطعت القيام بها كالمعتاد<br>(0) No, I have been coping as well as ever	☐كلا لقد استطعت القيام بها في معظم الاحيان<br>(1) No, most of the time I have coped quite will

21. لقد كنت غير ســعيدة لدرجة ان لدي صــعوبة في النوم*

I have been so unhappy that I have had difficulty sleeping*

☐كلا مطلقا<br>(0) No, not at all	☐ليس اغلب الاحيان<br>(1) Not very often	☐نعم في بعض الاحيان<br>(2) Yes, sometimes	☐نعم في معظم الاحيان<br>(3) Yes, most of the time

22. شعرت بانني حزينة وبائسة*

I have felt sad or miserable*

☐كلا مطلقا	☐كلا ليس اغلب الاحيان	☐نعم في اغلب الاحيان	☐نعم في معظم الاحيان
(0) No, not at all	(1) Not very often	(2) Yes, quite often	(3) Yes, most of the time

23. لقد كنت غير سعيدة للغاية لدرجة اني كنت ابكي*

I have been so unhappy that I have been crying*

☐كلا ابدا<br>(0) No, not at all	☐فقط من وقت لأخر<br>(1) Only occasionally	☐نعم في اغلب الاحيان<br>(2) Yes, quite often	☐نعم في معظم الاحيان<br>(3) Yes, most of the time

24. لقد خطرت لي فكرة الحاق الاذى بنفسي*

The thought of harming myself has occurred to me*

☐كلا ابدا<br>(0) Never	☐نادرا<br>(1) Hardly ever	☐نعم في بعض الاحيان<br>(2) Sometimes	☐نعم في احوال كثيرة<br>(3) Yes, quite often

Institutional Review Board · Hamad Medical Corporation · Doha · Qatar · Hamad

MRC-01-20-1129 Validity: 04 04 2020 - 12 07 2020 E-stamped 05 Apr 2021

Total Score of EPDS…

المجموع: ....

Research Questions Related to COVID-19 Phobia	"strongly disagree," لا أوافق بشدة	"disagree," لا أوافق	"neither agree nor disagree," لا اعلم	"agree أوافق,"	"strongly agree أوافق بشدة
4. I am most afraid of coronavirus-19. أخشى أكثر من فيروس التاجي -19.	0	1	2	3	4
2.It makes me uncomfortable to think about coronavirus-19. أشعر بعدم الارتياح للتفكير في فيروس كورونا -19	0	1	2	3	4
3.My hands become clammy when I think about coronavirus-19. أصبحت يدي رطبة عندما أفكر في فيروس التاجي -19.	0	1	2	3	4
4.I am afraid of losing my life because of coronavirus-19. أخشى أن أفقد حياتي بسبب فيروس كورونا -19.	0	1	2	3	4
5.When watching news and stories about coronavirus-19 on social media, I become nervous or anxious. عندما أشاهد الأخبار والقصص حول فيروس التاجي -19 على وسائل التواصل الاجتماعي ، أصبت بالتوتر أو القلق.	0	1	2	3	4
6.I cannot sleep because I'm worrying about getting coronavirus-19.	0	1	2	3	4

Institutional Review Board Hamad Medical Corporation - Doha - Qatar Hamad

MRC-01-20-1129 Validity: 04 04 2020 - 12 07 2020 E-stamped 05 Apr 2021

لا أستطيع النوم لأنني قلق بشأن الإصابة بالفيروس التاجي -19					
7.My heart races or palpitates when I think about getting coronavirus-19.<br><br>يسرع قلبي أو يخفق عندما أفكر في الإصابة بالفيروس التاجي -19.	0	1	2	3	4

Institutional Review Board Hamad Medical Corporation - Doha - Qatar Hamad
MRC-01-20-1129 Validity: 04 04 2020 - 12 07 2020 E-stamped 05 Apr 2021

Structured Questionnaire for T2 to T5 *(the participant is postpartum)*

Circle the number of session:

T4

Appendix III

Structured Questionnaire- Video telecommunication

تاريخ جمع البيانات	عدم المشاركة	اسم جامع البيانات	رقم المشاركة
Date	Drop out	Data Collector Name	Participant Number
………/……. / …	....	....	....

((بما انك حامل وصحتك تهمنا نتمنى ان ناخذ من وقتك خمسة عشر دقيقة لنسالك بعض الاسئلة .القسم الأول سيشمل مقابلة بحيث يسالك الباحث بعض الاسئلة. اما القسم الثاني فتقرير ذاتي تمليه المشتركة .))

Congratulation on your baby! As you are in your postpartum period and your health status matters to us, we would like to ask you some questions. It will include face-to-face interview-based questionnaire and standard tools through the video telecommunication. It will not take more than 15 minutes.

Section I: Questionnaire / الجزء الاول: الاستبيان

يستند هذا الاستبيان على مقابلة تقوم بالتحقيق في بعض الجوانب المتعلقة بحياتك الاجتماعية والديموغرافية والاقتصادية. كما ستسالين عن تاريخك الطبي وعادات أسلوب حياتك ودعم زوجك والمساعدة التي تتوقعينها بعد الولادة.

This is an interview-based questionnaire, going to investigate some aspects related to socio demographical factors, economic factors, medical history and life style habit. Additionally, you will be asked about your partner and the expected postpartum support .

MRC-01-20-1129 Validity: 04 04 2020 - 12 07 2020 E-stamped 05 Apr 2021

25. Mother health: Any health complication…………..
26. Baby health: any complication….

س –عوامل نمط الحياة الحالية

H-Life style habits

Do you do regular sport different from your house activity?

42) هل تمارســـين الرياضـــة المنتظمة (باســـتثناء العمل المنزلي)؟

No لا☐ Yes نعم☐

How much time you spend on sport activities?

43) كم من الوقت تقضيه في الأنشطة الرياضية؟

>20min اكثر من عشرين دقيقة☐ <20 min اقل من عشرين دقيقة☐

How many times per week do you do sport?

44) كم مرة في الأسبوع تقومين بالرياضة؟

اكثر من مرتين في الأسبوع☐ > 2 time/week

مرتين في الأسبوع☐ 2 time/week

اقل من مرة في الأسبوع☐ < 1 time/week.

Fitness Score

45) مؤشر النشاط البدني:

☐ المؤشر صفر: غير لائق بدنيا: القيام بنشاط بدني أقل من مرة أو مرتين في الأسبوع ولمدة زمنية تقل عن 20 دقيقة.
☐ المؤشر واحد: نشيط :أ داء النشاط البدني مرة واحدة إلى مرتين في الأسبوع لمدة 20 دقيقة ، أو أكثر من مرتين في الأسبوع لمدة أقل من 20 دقيقة
☐ المؤشر اثنين : لائق بدنيا أداء النشاط البدني أكثر من مرتين في الأسبوع لأكثر من 20 دقيقة

☐ *Not fit*: perform physical activity less than once or twice a week and for a period of less than 20 minutes (zero index score)
☐ *Active*: perform physical activity once to twice a week for 20 minutes, or more than twice a week for less than 20 minutes have a physical activity index of one
☐ *Fit*: perform physical activity more than twice a week for more than 20 minutes

Institutional Review Board Hamad Medical Corporation · Doha · Qatar

MRC-01-20-1129 Validity: 04 04 2020 - 12 07 2020 E-stamped 05 Apr 2021

(46 هل تدخنين (الارغيلة او السيجار)؟

Do you smoke? (Hubble-bubble, tobacco)

☐لم ادخن أبدًا

Never smoked

☐ادخن كل يوم

Every day smoker

☐ادخن حاليا ولكن ليس كل يوم

Current smoker but not every day

☐لا ادخن حالياً ولكن كنت ادخن سابقا (على الاقل 100 سيجارة في حياتي)

Former smoker (smoked at least 100 cigarettes but currently not smoking)

(47 هل تشربين الخمر؟

Do you drink alcohol?

☐لا ابدا

No, never had alcohol before.

☐حاليا لا اشرب ولكن كنت اشرب سابقا قبل الحمل

I don’t drink now but I used to drink before pregnancy.

☐نعم اشرب حاليا خلال الحمل

Yes, I am drinking alcohol during my pregnancy.

I- Stressful life events

ش –أحداث الحياة المجهدة

(48 هـل تواجهين أي حـدث مرهق او مجهد او ازمـة في حياتك حاليا؟

Do you consider yourself living stressful life event?

☐نعم

Yes

☐لا

No

(49 اذا كان الجواب نعم ما هي الازمة؟....................

If yes, kindly mention your stressful life event:

(50 الى اي حد انت متوترة بخصوص جنس المولود؟

MRC-01-20-1129 Validity: 04 04 2020 - 12 07 2020 E-stamped 05 Apr 2021

To what extent have you been stressed about specific baby gender?

☐لا على الإطلاق<br>Not at All	☐إلى حد صغير<br>Very Little	☐بعض الاحيان<br>Somewhat	☐إلى حد كبير<br>To a Great Extent

27. الى أي حد تواجهين ضائقة مالية؟

How often do you face financial distress?

☐دائما<br>Always (3)	☐ أحيانا<br>Sometimes (2)	☐ نادرًا<br>(1) Rarely	☐أبدا<br>Never (0)

القسم الثاني
Section II: Standard Tools

Tool (1): Perinatal depression screening tool (EPDS)/اكتئاب الحمل

نرجو ان تختاري الجواب الأنسب الذي يعبر عن مشاعرك خلال الايام السبعة الماضية وليس مشاعرك اليوم فحسب (جواب واحد فقط) :

Please choose which comes closest to how you have felt IN THE PAST 7 DAYS, not just how you feel today (Only choose one answer):

Here is an example already completed

اليك مثل و قد اكمل:

I have felt Happy:

لقد شعرت بانني سعيدة:

☐كلا مطلقا<br>(3) Not at all	☐ كلا ليس في احوال كثيرة<br>(2) No, Not very often	☒ نعم معظم الاوقات<br>(1) Yes, some of the time	☐ نعم كل الاوقات<br>(0) Yes, most of the time

This would mean that: I have felt happy some of the time during the past week not just how you feel today. Please complete other questions in the same way:

و هذايعني لقد شعرت بانني سعيدة معظم الوقت خلال الاسبوع الماضي وليس مشاعرك اليوم فحسب

الرجاء ان تكملي الاسالة الاخرى بالطريقة ذاتها

In the past 7 days:

خلال الايام السبعة الماضية:

MRC-01-20-1129 Validity: 04 04 2020 - 12 07 2020 E-stamped 05 Apr 2021

27. لقد تمكنت من الضحك و رؤية الجانب المضحك من الأمور

I have been able to laugh and see the funny side of things as much as I always could

☐كلا مطلقا<br><br>(3) Not at all	☐قطعا ليس بالمقدار نفسه الان<br><br>(2) Definitely not so much now	☐ليس تماما بالمقدار نفسه الان<br><br>(1) Not quite so much now.	☐بالمقدار الذي استطعته دائما<br><br>(0) As much as I always could

28. لقد تطلعت الى الامور بمتعة

I have looked forward with enjoyment to things

☐نادرا<br><br>(3) Hardly at all	☐قطعا اقل مما اعتدته<br><br>(2) Definitely less than I used to	☐نوع ما اقل مما اعتدته<br><br>(1) Rather less than I used to	☐بالمقدار نفسه مثل اي وقت مضى<br><br>(4) As much as I ever did

29. لقد لمت نفسي بدون لزوم عندما سارت الامور على غير ما يرام*

I have blamed myself unnecessarily when things went wrong. *

☐كلا ابدا<br><br>(0) No, never	☐ليس اغلب الاحيان<br><br>(1) Not very often	☐نعم في بعض الاحيان<br><br>(2) Yes, some of the time	☐نعم في معظم الاحيان<br><br>(3) Yes, most of the time

30. لقد كنت قلقة ومشغولة البال من دون سبب وجيه

I have been anxious or worried for no good reasons

☐نعم اغلب الأحيان<br><br>(3) Yes, very often	☐نعم في بعض الاحيان<br><br>(2) Yes, sometimes	☐نادرا<br><br>(1) Hardly, ever	☐كلا مطلقا<br><br>(0) No, not at all

31. لقد شعرت بالخوف والذعر من دون سبب وجيه*

I have felt scared or panicky for no very good reason*

☐كلا مطلقا	☐كلا ليس كثيرا	☐نعم في بعض الاحيان	☐نعم اكثر الاحيان
(0) No, not at all	(1) No, not much	(2) Yes, sometimes	(3) Yes, quite a lot

MRC-01-20-1129 Validity: 04 04 2020 - 12 07 2020 E-stamped 05 Apr 2021

Things have been getting on top of me*

32. تراكمت الاعمال علي فلم استطع القيام بها*

☐نعم في بعض الاحيان لم استطع القيام بها كالمعتاد<br>(2) Yes, sometimes I haven't been coping as well as usual	☐نعم في معظم الاحيان لم استطع القيام بها مطلقا<br>(3) Yes, most of the time I haven't been able to cope at all
☐كلا لقد استطعت القيام بها كالمعتاد<br>(0) No, I have been coping as well as ever	☐كلا لقد استطعت القيام بها في معظم الاحيان<br>(1) No, most of the time I have coped quite will

I have been so unhappy that I have had difficulty sleeping*

33. لقد كنت غير سعيدة لدرجة ان لدي صعوبة في النوم*

☐كلا مطلقا<br>(0) No, not at all	☐ليس اغلب الاحيان<br>(1) Not very often	☐نعم في بعض الاحيان<br>(2) Yes, sometimes	☐نعم في معظم الاحيان<br>(3) Yes, most of the time

I have felt sad or miserable*

34. شعرت بانني حزينة وبائسة*

☐كلا مطلقا	☐كلا ليس اغلب الاحيان	☐نعم في اغلب الاحيان	☐نعم في معظم الاحيان
(0) No, not at all	(1) Not very often	(2) Yes, quite often	(3) Yes, most of the time

I have been so unhappy that I have been crying*

35. لقد كنت غير سعيدة للغاية لدرجة اني كنت ابكي*

☐كلا ابدا<br>(0) No, not at all	☐فقط من وقت لأخر<br>(1) Only occasionally	☐نعم في اغلب الاحيان<br>(2) Yes, quite often	☐نعم في معظم الاحيان<br>(3) Yes, most of the time

The thought of harming myself has occurred to me*

36. لقد خطرت لي فكرة الحاق الاذى بنفسي*

☐كلا ابدا<br>(0) Never	☐نادرا<br>(1) Hardly ever	☐نعم في بعض الاحيان<br>(2) Sometimes	☐نعم في احوال كثيرة<br>(3) Yes, quite often

MRC-01-20-1129 Validity: 04 04 2020 - 12 07 2020 E-stamped 05 Apr 2021

Total Score of EPDS…

المجموع: ....

Research Questions Related to COVID-19 Phobia	“strongly disagree,” لا أوافق بشدة	“disagree,” لا أوافق	“neither agree nor disagree,” لا اعلم	“agree أوافق,”	“strongly agree أوافق بشدة
5. I am most afraid of coronavirus-19. أخشى أكثر من فيروس التاجي -19.	0	1	2	3	4
2.It makes me uncomfortable to think about coronavirus-19. أشعر بعدم الارتياح للتفكير في فيروس كورونا -19	0	1	2	3	4
3.My hands become clammy when I think about coronavirus-19. أصبحت يدي رطبة عندما أفكر في فيروس التاجي -19.	0	1	2	3	4
4.I am afraid of losing my life because of coronavirus-19. أخشى أن أفقد حياتي بسبب فيروس كورونا -19.	0	1	2	3	4
5.When watching news and stories about coronavirus-19 on social media, I become nervous or anxious. عندما أشاهد الأخبار والقصص حول فيروس التاجي -19 على وسائل التواصل الاجتماعي ، أصبت بالتوتر أو القلق.	0	1	2	3	4
6.I cannot sleep because I’m worrying about getting coronavirus-19.	0	1	2	3	4

Institutional Review Board Hamad Medical Corporation - Doha - Qatar Hamad

MRC-01-20-1129 Validity: 04 04 2020 - 12 07 2020 E-stamped 05 Apr 2021

لا أستطيع النوم لأنني قلق بشأن الإصابة بالفيروس التاجي -19					
7.My heart races or palpitates when I think about getting coronavirus-19.<br><br>يسرع قلبي أو يخفق عندما أفكر في الإصابة بالفيروس التاجي -19.	0	1	2	3	4

MRC-01-20-1129 Validity: 04 04 2020 - 12 07 2020 E-stamped 05 Apr 2021

Structured Questionnaire for T2 to T5 *(the participant is postpartum)*

Circle the number of session:

T5

Appendix III

Structured Questionnaire- Video telecommunication

تاريخ جمع البيانات	عدم المشاركة	اسم جامع البيانات	رقم المشاركة
Date	Drop out	Data Collector Name	Participant Number
………/……. / …	....	....	....

((بما انك حامل وصحتك تهمنا نتمنى ان ناخذ من وقتك خمسة عشر دقيقة لنسالك بعض الاسئلة .القسم الأول سيشمل مقابلة بحيث يسالك الباحث بعض الاسئلة. اما القسم الثاني فتقرير ذاتي تمليه المشتركة .))

Congratulation on your baby! As you are in your postpartum period and your health status matters to us, we would like to ask you some questions. It will include face-to-face interview-based questionnaire and standard tools through the video telecommunication. It will not take more than 15 minutes.

Section I: Questionnaire / الجزء الاول: الاستبيان

يستند هذا الاستبيان على مقابلة تقوم بالتحقيق في بعض الجوانب المتعلقة بحياتك الاجتماعية والديموغرافية والاقتصادية. كما ستسالين عن تاريخك الطبي وعادات أسلوب حياتك ودعم زوجك والمساعدة التي تتوقعينها بعد الولادة.

This is an interview-based questionnaire, going to investigate some aspects related to socio demographical factors, economic factors, medical history and life style habit. Additionally, you will be asked about your partner and the expected postpartum support .

MRC-01-20-1129 Validity: 04 04 2020 - 12 07 2020 E-stamped 05 Apr 2021

37. Mother health: Any health complication……………
38. Baby health: any complication….

س –عوامل نمط الحياة الحالية

H-Life style habits

(51 هل تمارسـين الرياضـة المنتظمة (باستثناء العمل المنزلي)؟

Do you do regular sport different from your house activity?

☐نعم Yes ☐لا No

(52 كم من الوقت تقضيه في الأنشطة الرياضية؟

How much time you spend on sport activities?

☐اقل من عشرين دقيقة <20 min ☐اكثر من عشرين دقيقة >20min

(53 كم مرة في الأسبوع تقومين بالرياضة؟

How many times per week do you do sport?

☐اقل من مرة في الأسبوع < 1 time/week.

☐مرتين في الأسبوع 2 time/week

☐اكثر من مرتين في الأسبوع > 2 time/week

(54 **مؤشر النشاط البدني:**

Fitness Score

- ☐ المؤشر صفر: غير لائق بدنيا: القيام بنشاط بدني أقل من مرة أو مرتين في الأسبوع ولمدة زمنية تقل عن 20 دقيقة.
- ☐ المؤشر واحد: نشيط :أ داء النشاط البدني مرة واحدة إلى مرتين في الأسبوع لمدة 20 دقيقة ، أو أكثر من مرتين في الأسبوع لمدة أقل من 20 دقيقة
- ☐ المؤشر اثنين : لائق بدنيا أداء النشاط البدني أكثر من مرتين في الأسبوع لأكثر من 20 دقيقة

- ☐ *Not fit*: perform physical activity less than once or twice a week and for a period of less than 20 minutes (zero index score)
- ☐ *Active*: perform physical activity once to twice a week for 20 minutes, or more than twice a week for less than 20 minutes have a physical activity index of one
- ☐ *Fit*: perform physical activity more than twice a week for more than 20 minutes

Institutional Review Board Hamad Medical Corporation · Doha · Qatar

MRC-01-20-1129 Validity: 04 04 2020 - 12 07 2020 E-stamped 05 Apr 2021

(55 هل تدخنين (الارغيلة او السيجار)؟

Do you smoke? (Hubble-bubble, tobacco)

☐لم ادخن أبدًا

Never smoked

☐ادخن كل يوم

Every day smoker

☐ادخن حاليا ولكن ليس كل يوم

Current smoker but not every day

☐لا ادخن حالياً ولكن كنت ادخن سابقا (على الاقل 100 سيجارة في حياتي)

Former smoker (smoked at least 100 cigarettes but currently not smoking)

(56 هل تشربين الخمر؟

Do you drink alcohol?

☐لا ابدا

No, never had alcohol before.

☐حاليا لا اشرب ولكن كنت اشرب سابقا قبل الحمل

I don't drink now but I used to drink before pregnancy.

☐نعم اشرب حاليا خلال الحمل

Yes, I am drinking alcohol during my pregnancy.

I- Stressful life events

ش –أحداث الحياة المجهدة

(57 هـل تواجهين أي حـدث مرهق او مجهد او ازمـة في حياتك حاليا؟

Do you consider yourself living stressful life event?

☐نعم

Yes

☐لا

No

(58 اذا كان الجواب نعم ما هي الازمة؟....................

If yes, kindly mention your stressful life event:

(59 الى اي حد انت متوترة بخصوص جنس المولود؟

MRC-01-20-1129 Validity: 04 04 2020 - 12 07 2020 E-stamped 05 Apr 2021

To what extent have you been stressed about specific baby gender?

☐لا على الإطلاق<br>Not at All	☐إلى حد صغير<br>Very Little	☐بعض الاحيان<br>Somewhat	☐إلى حد كبير<br>To a Great Extent

---

28. الى أي حد تواجهين ضائقة مالية؟

How often do you face financial distress?

☐دائما<br>Always (3)	☐ أحيانا<br>Sometimes (2)	☐ نادرًا<br>(1) Rarely	☐أبدا<br>Never (0)

القسم الثاني

Section II: Standard Tools

Tool (1): Perinatal depression screening tool (EPDS)/اكتئاب الحمل

نرجو ان تختاري الجواب الأنسب الذي يعبر عن مشاعرك خلال الايام السبعة الماضية وليس مشاعرك اليوم فحسب (جواب واحد فقط) :

Please choose which comes closest to how you have felt IN THE PAST 7 DAYS, not just how you feel today (Only choose one answer):

Here is an example already completed

اليك مثل و قد اكمل:

I have felt Happy:

لقد شعرت بانني سعيدة:

☐كلا مطلقا<br>(3) Not at all	☐ كلا ليس في احوال كثيرة<br>(2) No, Not very often	☒ نعم معظم الاوقات<br>(1) Yes, some of the time	☐ نعم كل الاوقات<br>(0) Yes, most of the time

This would mean that: I have felt happy some of the time during the past week not just how you feel today. Please complete other questions in the same way:

و هذايعني لقد شعرت بانني سعيدة معظم الوقت خلال الاسبوع الماضي وليس مشاعرك اليوم فحسب

الرجاء ان تكملي الاسالة الاخرى بالطريقة ذاتها

In the past 7 days:

خلال الايام السبعة الماضية:

39.لقد تمكنت من الضحك و رؤية الجانب المضحك من الأمور

I have been able to laugh and see the funny side of things as much as I always could

☐كلا مطلقا<br>(3) Not at all	☐قطعا ليس بالمقدار نفسه الان<br>(2) Definitely not so much now	☐ليس تماما بالمقدار نفسه الان<br>(1) Not quite so much now.	☐بالمقدار الذي استطعته دائما<br>(0) As much as I always could

40.لقد تطلعت الى الامور بمتعة

I have looked forward with enjoyment to things

☐نادرا<br>(3) Hardly at all	☐قطعا اقل مما اعتدته<br>(2) Definitely less than I used to	☐نوع ما اقل مما اعتدته<br>(1) Rather less than I used to	☐بالمقدار نفسه مثل اي وقت مضى<br>(5) As much as I ever did

41.لقد لمت نفسي بدون لزوم عندما سارت الامور على غير ما يرام*

I have blamed myself unnecessarily when things went wrong. *

☐كلا ابدا<br>(0) No, never	☐ليس اغلب الاحيان<br>(1) Not very often	☐نعم في بعض الاحيان<br>(2) Yes, some of the time	☐نعم في معظم الاحيان<br>(3) Yes, most of the time

42.لقد كنت قلقة ومشغولة البال من دون سبب وجيه

I have been anxious or worried for no good reasons

☐نعم اغلب الأحيان<br>(3) Yes, very often	☐نعم في بعض الاحيان<br>(2) Yes, sometimes	☐نادرا<br>(1) Hardly, ever	☐كلا مطلقا<br>(0) No, not at all

43. لقد شعرت بالخوف والذعر من دون سبب وجيه*

I have felt scared or panicky for no very good reason*

☐كلا مطلقا<br>(0) No, not at all	☐كلا ليس كثيرا<br>(1) No, not much	☐نعم في بعض الاحيان<br>(2) Yes, sometimes	☐نعم اكثر الاحيان<br>(3) Yes, quite a lot

MRC-01-20-1129 Validity: 04 04 2020 - 12 07 2020 E-stamped 05 Apr 2021

Things have been getting on top of me*

44. تراكمت الاعمال علي فلم استطع القيام بها*

☐نعم في بعض الاحيان لم استطع القيام بها كالمعتاد<br>(2) Yes, sometimes I haven't been coping as well as usual	☐نعم في معظم الاحيان لم استطع القيام بها مطلقا<br>(3) Yes, most of the time I haven't been able to cope at all
☐كلا لقد استطعت القيام بها كالمعتاد<br>(0) No, I have been coping as well as ever	☐كلا لقد استطعت القيام بها في معظم الاحيان<br>(1) No, most of the time I have coped quite will

I have been so unhappy that I have had difficulty sleeping*

45. لقد كنت غير سعيدة لدرجة ان لدي صعوبة في النوم*

☐كلا مطلقا<br>(0) No, not at all	☐ليس اغلب الاحيان<br>(1) Not very often	☐نعم في بعض الاحيان<br>(2) Yes, sometimes	☐نعم في معظم الاحيان<br>(3) Yes, most of the time

I have felt sad or miserable*

46. شعرت بانني حزينة وبائسة*

☐كلا مطلقا	☐كلا ليس اغلب الاحيان	☐نعم في اغلب الاحيان	☐نعم في معظم الاحيان
(0) No, not at all	(1) Not very often	(2) Yes, quite often	(3) Yes, most of the time

I have been so unhappy that I have been crying*

47. لقد كنت غير سعيدة للغاية لدرجة اني كنت ابكي*

☐كلا ابدا<br>(0) No, not at all	☐فقط من وقت لأخر<br>(1) Only occasionally	☐نعم في اغلب الاحيان<br>(2) Yes, quite often	☐نعم في معظم الاحيان<br>(3) Yes, most of the time

The thought of harming myself has occurred to me*

48. لقد خطرت لي فكرة الحاق الاذى بنفسي*

☐كلا ابدا<br>(0) Never	☐نادرا<br>(1) Hardly ever	☐نعم في بعض الاحيان<br>(2) Sometimes	☐نعم في احوال كثيرة<br>(3) Yes, quite often

Institutional Review Board Hamad Medical Corporation · Doha · Qatar Hamad

MRC-01-20-1129 Validity: 04 04 2020 - 12 07 2020 E-stamped 05 Apr 2021

Total Score of EPDS…

المجموع: ....

Research Questions Related to COVID-19 Phobia	“strongly disagree,” لا أوافق بشدة	“disagree,” لا أوافق	“neither agree nor disagree,” لا اعلم	“agree أوافق,”	“strongly agree أوافق بشدة
6. I am most afraid of coronavirus-19. أخشى أكثر من فيروس التاجي -19.	0	1	2	3	4
2.It makes me uncomfortable to think about coronavirus-19. أشعر بعدم الارتياح للتفكير في فيروس كورونا -19	0	1	2	3	4
3.My hands become clammy when I think about coronavirus-19. أصبحت يدي رطبة عندما أفكر في فيروس التاجي -19.	0	1	2	3	4
4.I am afraid of losing my life because of coronavirus-19. أخشى أن أفقد حياتي بسبب فيروس كورونا -19.	0	1	2	3	4
5.When watching news and stories about coronavirus-19 on social media, I become nervous or anxious. عندما أشاهد الأخبار والقصص حول فيروس التاجي -19 على وسائل التواصل الاجتماعي ، أصبت بالتوتر أو القلق.	0	1	2	3	4
6.I cannot sleep because I’m worrying about getting coronavirus-19.	0	1	2	3	4

MRC-01-20-1129 Validity: 04 04 2020 - 12 07 2020 E-stamped 05 Apr 2021

لا أستطيع النوم لأنني قلق بشأن الإصابة بالفيروس التاجي -19					
7.My heart races or palpitates when I think about getting coronavirus-19.<br><br>يسرع قلبي أو يخفق عندما أفكر في الإصابة بالفيروس التاجي -19.	0	1	2	3	4
