# Supplementary material for: Maternal low-intensity psychosocial telemental interventions in response to COVID-19 in Qatar: study protocol for a randomized controlled trial
Source: Trials. 2021 Jun 7;22:382. doi: 10.1186/s13063-021-05339-w (PMC8181539; doi:10.1186/s13063-021-05339-w)
Supplement: Supplementary file 2 — Additional file 2. Screening sheet. [file 13063_2021_5339_MOESM2_ESM.pdf]

Appendix-II

Screening Phase Questions- Asked following telephone script:

Service: Telemental Health Consultation Phone Call.

الخدمة: مكالمة هاتفية للاستشارة النفسية الصحية الهاتفية

To include participant, she must verbally consent as well as to answer (No) to first two questions and (Yes) to third question.

للمشاركة ، يجب أن توافق شفهيا وكذلك يجب ان تكون الإجابة (لا) على أول سؤالين و (نعم) على السؤال الثالث.

1) Are you following up with any psychiatric hospital or doctor? Yes / No

1) هل تتابعين مع أي مستشفى أو طبيب نفسي؟ نعم / لا

2) Are you currently receiving any medications related to mental health more than 6 times in any year (Drugs like Valium, Xanax, Ativan, Klonopin, Ambien, Sonata, or Lunesta)? Yes / No

هل تتلقين حاليًا أي أدوية تتعلق بالصحة النفسية أكثر من 6 مرات في أي عام (أدوية مثل Valium, Xanax, Ativan,

Klonopin, Ambien, Sonata, or Lunesta)؟ نعم / لا

3) Download the Vsee clinic icon (green colour-symbol C) on the phone? Yes/ No

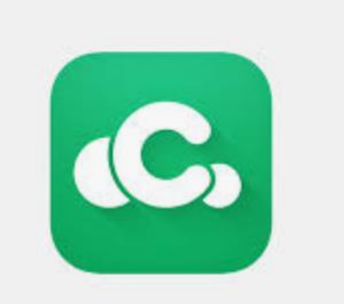

هل تم تحميل ال vsee التطبيق (باللون الاخضر -الاشارة C) على التلفون؟ نعم / لا

Conclusion	☐ Fulfilled Inclusion criteria<br>☐ Fulfilled Exclusion criteria
النتيجة	☐ تضمن<br>☐ استبعاد
