# Supplementary material for: Maternal low-intensity psychosocial telemental interventions in response to COVID-19 in Qatar: study protocol for a randomized controlled trial
Source: Trials. 2021 Jun 7;22:382. doi: 10.1186/s13063-021-05339-w (PMC8181539; doi:10.1186/s13063-021-05339-w)
Supplement: Supplementary file 3 — Additional file 3. Consent form. [file 13063_2021_5339_MOESM3_ESM.pdf]

Telephone Script نسخة هاتفية

Each potential participant will be contacted a maximum of three times (each attempt separated from the other by 5 minutes), before withdrawing their name from the potential participant list.

سيتم الاتصال بكل مشارك محتمل ثلاث مرات كحد أقصى (كل محاولة منفصلة عن الأخرى بمقدار 5 دقائق) ، قبل سحب اسمه من قائمة المشاركين المحتملين.

Dear participant (participant name), good day. You are being contacted today to participate in a study project title is ***Low intensity psychosocial Interventions in pregnant women in Response to Covid-19: Maternal Mental Health Matters' An interventional study'***

عزيزتي المشاركة (اسم المشاركة) ، يوم جيد. يتم الاتصال بك اليوم للمشاركة في دراسة عن تدخلات نفسية واجتماعية منخفضة الشدة لدى الحوامل استجابة لـ Covid-19: مسائل الصحة النفسية للأمهات "دراسة تداخلية"

If participant answers with a **no**, the data collector will apologize to her for the disturbance and hang up. If possible, will ask about refusal reason.

إذا أجابت المشاركة برفض ، فسوف يعتذر جامع البيانات لها عن الإزعاج ويغلق المكالمة. إذا أمكن ، سيسأل عن سبب الرفض

Refusal Reason…….

سبب الرفض.......

If she said yes:

اذا اجابت نعم:

This research is about Perinatal women (pregnant and in their postpartum) during COVID 19 epidemic could be at risk of severe form of phobia, psychological distress and anxiety in pregnancy than the non-perinatal population Mental health disorders (anxiety, depression and phobias) showed to affect negatively the expecting mothers and their offspring, increasing the odds of pre-term delivery.

هذا البحث عن النساء الحوامل والاتي قمن بالولادة خلال وباء COVID 19 في خطر من شكل شديد من الرهاب والضيق النفسي والقلق في هذه الفترة. فقد اظهرت الدراسات ان الاضطرابات الصحية العقلية (القلق والاكتئاب والرهاب) تؤثر سلبا على الأمهات المتوقع وضعهم وذريتهم، مما يزيد من احتمالات الولادة قبل الأوان.

The aim of this study to provide WHO low psychosocial intervention that may help u cope with all the stress associated with Covid-19.

تهدف هذه الدراسة إلى توفير تدخل نفسي اجتماعي منخفض لمنظمة الصحة العالمية قد يساعدك على التعامل مع جميع الضغوط المرتبطة بـ Covid-19
.

The purpose of this study is to provide perinatal women (pregnant and in their early postpartum) low psychosocial intervention, that may decrease the burden of perinatal mental illnesses on the expecting mothers, children, health care system and eventually better quality of life during this pandemic

.الغرض من إجراء دراسة البحث توفير تدخل نفسي اجتماعي منخفض للنساء الحوامل (في فترة الحمل وبعد الولادة المبكرة) ، مما قد يقلل من عبء الأمراض العقلية في الفترة المحيطة بالولادة على الأمهات والأطفال ، ونظام الرعاية الصحية ، وفي نهاية المطاف نوعية حياة أفضل خلال فترة الوباء.

You are invited to participate and consent As you are pregnant women and or early postpartum period.

تم توجيه الدعوة لك للمشاركة في هذا البحث بما انك امرأة حامل أو في فترة ما بعد الولادة .

Accepting or rejecting to participate will not affect your usual care. You will still receive all your usual care. What is different is that if you accept, we will ask you some questions related to mental health through phone and we will arrange with you six low psychosocial support sessions provided through video-teleconsultation.

Randomization: Probability Sampling Technique (simple random selection) will be employed to select randomly through *Automated Random Number Generator* the participants. Random allocation of the participants will be done into the intervention and control group.

لن يؤثر قبول أو رفض المشاركة على رعايتك المعتادة. ستظل تتلقى كل الرعاية المعتادة الخاصة بك. ما هو مختلف هو أنه إذا قبلت ، فسوف نطرح عليك بعض الأسئلة المتعلقة بالصحة العقلية عبر الهاتف وسنرتب معك ست جلسات دعم نفسي اجتماعي منخفضة. المقدمة من خلال استشارة الفيديو

التوزيع العشوائي: سيتم استخدام تقنية أخذ العينات الاحتمالية (التحديد العشوائي البسيط) للاختيار عشوائيًا من خلال مولد الرقم العشوائي الآلي المشاركين. سيتم تخصيص المشاركين بشكل عشوائي في مجموعة التدخل والسيطرة.

There are no risks associated with the participation in this research. Completing the telephone script will consume up to 30 minutes and each video teleconsultation will take 45 min. We hope that this will not cause any discomfort or inconvenience.

لا توجد مخاطر مرتبطه بالمشاركة في هذا البحث. سيستغرق ملء الاستبيان ما يصل إلى45 دقيقة. نأمل أن هذا لن يسبب أي إزعاج.

When we feel it is necessary and likely that you need help, we will discuss with you and refer you for psychological counselling. Additionally, we will store all information you provide securely and will not make it available to anyone else.

عندما نشعر أنه من الضروري مساعدتك على الأرجح، سنناقش معك ونحيلك للحصول على المشورة النفسية.كما سنخزن جميع المعلومات التي تقدمها بأمان ولن نوفرها لأي شخص آخر.

In case you have any depression, anxiety or stress we will receive the appropriate support.

في حال كان لديك أي الاكتئاب أو القلق أو الإجهاد تحصلين على الدعم المناسب.

. We appreciate your participation in this study. Please note that we do not pay volunteers for participation.

نقدر مشاركتك في هذه الدراسة. يرجى العلم أننا لا ندفع للمتطوعين مقابل المشاركة

1-Verbal consent will be utilized, each participant will take her time to understand and approve informed consent.

1- سيتم استخدام الموافقة الشفهية، وستأخذ كل مشاركة وقتها للفهم والموافقة عليها.

2-Autonomy will be ensured were each participant will have free choice to participate or withdraw without affecting her treatment or health service she receives.

2- سيتم ضمان الاستقلال الذاتي إذا كان لكل مشارك حرية الاختيار للمشاركة أو الانسحاب دون التأثير على علاجها أو خدمة الرعاية الصحية التي تتلقاها.

3- Privacy will be addressed: No HC number or names will be identified at any time of the data collection. (HC numbers will be coded)

3- سيتم تناول الخصوصية: لن يتم تحديد أي رقم أو أسماء HC في أي وقت من عملية جمع البيانات. (سيتم ترميز أرقام HC)

For referral, the consents will be coded as well as the data collection tool to enable referral process.

وللتحويلات، سيتم ترميز الموافقات وكذلك أداة جمع البيانات لتمكين عملية التحويل.

There is no pressure on the prospective subject to participate in the study, that he/she is free to choose any of the treatment modalities offered and that there is no pressure on the participant to continue in the study even after enrollment. you have volunteered of your own free will to take part in this research. Please do not feel at any time under any obligation to take part in the research.

إقرار بعدم ممارسة ضغوط على المشارك للموافقة و انه حر في اختيار الوسيلة المناسبة للعلاج و انه غير ملزم بالاستمرار في الدراسة حتى بعد التسجيل

After agreeing to take part, if you change your mind or do not wish to take part in some aspects of the research you are free to choose or refuse to participate in any (or all) aspects of the study at any time.

لديك الحريه في اختيار أو رفض المشاركة في أي (أو جميع) جوانب الدراسة في أي وقت. إذا قررت عدم المشاركة. نحن نحترم حقك في المشاركة أو اختيار عدم المشاركة في البحث

You will be notified by researchers when the study is over or when your participation is not required. At the end of the study, we will explain how the results will be analyzed and whether we'll be able to share your results with you. Feel free to ask if you need to know more

سيتم إعلامك من قبل الباحثين عندما تنتهي الدراسة أو عندما لم تعد مشاركتك مطلوبة. في نهاية الدراسة ، سنشرح لك كيف سيتم تحليل النتائج وما إذا كنا سنكون قادرين على مشاركة نتائجك معك أم لا. لا تتردد في السؤال عما إذا كنت بحاجة إلى معرفة المزيد

In case you will like to provide feedback or raise concerns about the study, please contact:

HMC Institutional Review Board (HMC-IRB) Chair at 55546316HMC-IRB Office at 40256410 (from Sunday to Thursday between 7:00am-3:00pm) or email at irb@hamad.qa

Also you can contact

Dr. Sarah Naja

33615489

Snaja1@hamad.qa

في حال كنت ترغب في تقديم ملاحظات أو إثارة مخاوف بشأن الدراسة ، يرجى الاتصال بـ:

قسم البحوث السريرية ، مركز البحوث الطبية في مؤسسة حمد الطبية 55546316 مكتب IRB في 40256410

(من الأحد إلى الخميس بين 7:00 صباحًا و 3: 00 مساءً)

أو عبر البريد الإلكتروني irb@hamad.qa

ويمكنك الاتصال ب د سارة نجا 33615489

Snaja1@hamad.qa

	Time	Date	Initials
Telephone call (Attempt 1)			
Telephone call (Attempt 2)			
Telephone call (Attempt 3)			
Schedule the first video conference session 1 (To)			
Schedule the second video conference session 2 (T1)			
Schedule the third video conference Session 3 (T2)			
Schedule the fourth video conference Session 4(T3)			
Sechedule the fifth video conference session 5 (T4)			

**Schedule the Sixth video conference session 6 (T5)**			
